# Molecular epidemiology and clinical characteristics of herpangina children in Beijing, China: a surveillance study

**DOI:** 10.7717/peerj.9991

**Published:** 2020-10-15

**Authors:** Tian-Shuo Zhao, Juan Du, Hong-Jun Li, Yan Cui, Yaqiong Liu, Yanna Yang, Fuqiang Cui, Qing-Bin Lu

**Affiliations:** 1 Department of Laboratorial Science and Technology, School of Public Health, Peking University, Beijing, China; 2 Vaccine Research Center, School of Public Health, Peking University, Beijing, China; 3 Institute for Infectious Diseases and Endemic Diseases Prevention and Control, Beijing Tongzhou Center for Diseases Control and Prevention, Beijing, China

**Keywords:** Herpangina, Coxsackievirus, Children, Enterovirus

## Abstract

**Background:**

Herpangina is a highly infectious disease, which is usually prevalent in preschool children.

**Methods:**

This study analyzed the clinical and pathogenic characteristics of herpangina children to demonstrate the epidemiology of herpangina. Clinical manifestations, laboratory indicators and pharyngeal swabs were collected from children with herpangina who were monitored by Tongzhou Center for Disease Control and Prevention in Beijing, 2008. Utilizing pharyngeal swabs, virus extraction and amplification were performed for nucleotide identification and sequencing. The phylogenetic analysis was conducted based on all sequences amplified in this study and strains retrieved from GenBank.

**Results:**

Among 190 children with herpangina, 69.0% (131/190) were positive for enterovirus. Eight genotypes were identified, mainly including CV-A6 (39/131), CV-A4 (25/131), CV-A10 (24/131). The phylogenetic analysis showed one CV-A6 strain of Tongzhou was imported from Japan. CV-A10 strains were clustered into five groups (A-E). The dominant cluster of CV-A10 was Group E6 between 2009 and 2013, and converted to Group E5 after 2013. CV-A6 was the predominant pathogen causing herpangina in Tongzhou in 2018, followed by CV-A4 and CV-A10.

**Conclusions:**

The circulation of coxsackievirus had spatiotemporal cluster. In controlling the transmission of herpangina, the surveillance and reporting system should be enhanced.

## Introduction

Herpangina is a highly infectious disease, which was detected in 1924 (Cherry & Jahn, 1965) for the first time. It is usually prevalent in children under 5 years of age. Children with herpangina often present with sudden pharyngeal pain and fever, and multiple herpes or ulcer in the posterior pharyngeal, such as in the pharyngeal palatine arch, soft palate, uvula and tonsil (Hui, 2019; Park et al., 2012). The prognosis of herpangina is favorable, but a few of the severe children can be afflicted with encephalitis, aseptic meningitis, acute delayed paralysis (AFP), pulmonary edema, myocarditis and other complications, even death (Chang et al., 1999; Khetsuriani et al., 2006).

There have been multiple outbreaks of herpangina in Asian countries, including China, Thailand, Japan and Korea since 2009 (Chansaenroj et al., 2017; Li et al., 2016b; Park et al., 2012; Yamashita et al., 2005). Like hand, foot and mouth disease (HFMD), herpangina is mainly caused by enteroviruses (EVs) (Yao et al., 2017), particularly coxsackievirus (CV). The dominant EV serotypes were CV-A2 and CV-A5 in Korea in 2009(Park et al., 2012), CV-A10 and CV-A6 in France in 2010 (Mirand et al., 2012), CV-A2 and CV-A4 in Thailand in 2015 (Chansaenroj et al., 2017), respectively. A surveillance of 10,210 herpangina cases revealed that CV-A2 was the predominant EV serotype in 2015 in Zhejiang, China (Li et al., 2016c). The etiological spectrum of herpangina varied greatly, depending on the geographical locations, seasons and population susceptibility (Yamadera et al., 1991; Zhou et al., 2016).

According to the monitoring results of China National Statutory Infectious Disease Reporting System, HFMD was the predominant infectious disease in China in recent years (National Health Commission of China, 2019). As a disease that has an analogous pathogen of EVs to HFMD, herpangina has no impeccable monitoring or prevention strategy in most countries worldwide. The disease burden caused by herpangina may be highly underestimated. Moreover, there have been few molecular epidemiological studies on herpangina, especially in China. Therefore, this study analyzed the clinical and pathogenic characteristics of herpangina children to demonstrate the pathogen spectrum and the genetic evolution of the dominant EV serotypes.

## Materials & Methods

### Sample Collection

Between January and December of 2018, pediatric patients with clinical diagnosis of herpangina were monitored by Beijing Tongzhou Center for Disease Control and Prevention (CDC). Children with typically acute onset symptoms, including pharyngeal pain, fever, and herpes or ulcers on the palatine arch, soft palate, uvula and tonsil, but not on the hand, foot or trunk, were defined as herpangina and recruited in the study. The children without the pharyngeal swabs were excluded.

We collected the general demographic date, clinical characteristics and laboratory results for those children with herpangina met inclusive criteria for further analysis.

### Ethics statement

This study was approved by Peking University Institutional Review Board Office, with the approval number of IRB00001052-19005. All samples were collected through infectious disease surveillance system which was implemented based on oral informed consent to ensure compliance. Thus oral informed consents were obtained from parents or guardians before recruitment in this study.

### Virus extraction and identification

Pharyngeal swabs were collected for nucleotide identification among children with herpangina. RNA/DNA common extraction was performed with QIAamp MiniElute Virus Spin Kit (Cat.no.57704), according to the instructions of the manufacturer. The final extracts were stored in a 1.5 mL Rnase-Free fresh EP tube at −80 °C.

RNA/DNA extracts were amplified by a set of broad-spectrum primers for the 5′ untranslated region (5′UTR) of EVs. Next, specific primers based on VP1 genes were used for the amplification of specimens which were positive for CV-A4, CV-A6, CV-A10 by nested reverse transcription polymerase chain reaction (RT-PCR). All primers and reaction conditions selected in the experiment were designed according to the previous reports and were listed in Table S1. The amplicons products were visible on gels by electrophoresis, and positive products were sent for bidirectional sequencing using Sanger method.

### Sequence analysis

The identified nucleotides sequences were assembled utilizing Lasergene’s DNA SeqMan software (version 7.1.0, DNA Star Inc. Madison, WI, USA). Sequence alignment was conducted in nucleotides BLAST board based on NCBI between the obtained VP1 sequences in our study and homologous sequences from GenBank. All sequences identified in this study were submitted to the GenBank database (MN864893–MN864917 for CV-A4, MN864918–MN864953 for CV-A6 and MN864954–MN864977 for CV-A10).

All VP1 sequences of CV-A4, CV-A6 and CV-A10 in the GenBank were retrieved for the construction of the phylogenetic tree. The accessions and details of included strains were acquired from Table S2. The phylogenetic tree was constructed using the maximum-likelihood method with 1,000 bootstrap replications using MEGA (version 7.0.14, developed by Sudhir Kumar, Koichiro Tamura and Masatoshi Nei). Sequence identity matrixes between different branches were calculated by BioEdit (version 7.13, https://itservices.cas.unt.edu/software/bioedit725).

### Statistical analysis

Normally distributed continuous variables were described by mean ± standard deviation (SD), and the median and interquartile range (IQR) was used to describe non-normally distributed variables. Categorical variables were described with frequencies and proportions. Comparison between groups was performed by one-way analysis of variance, chi-square test/Fisher exact test or non-parametric test. All statistical steps were implemented by STATA (version 14.0, developed by statacorp LLC, USA).

## Results

### Demographics characteristics

**Figure 1:**
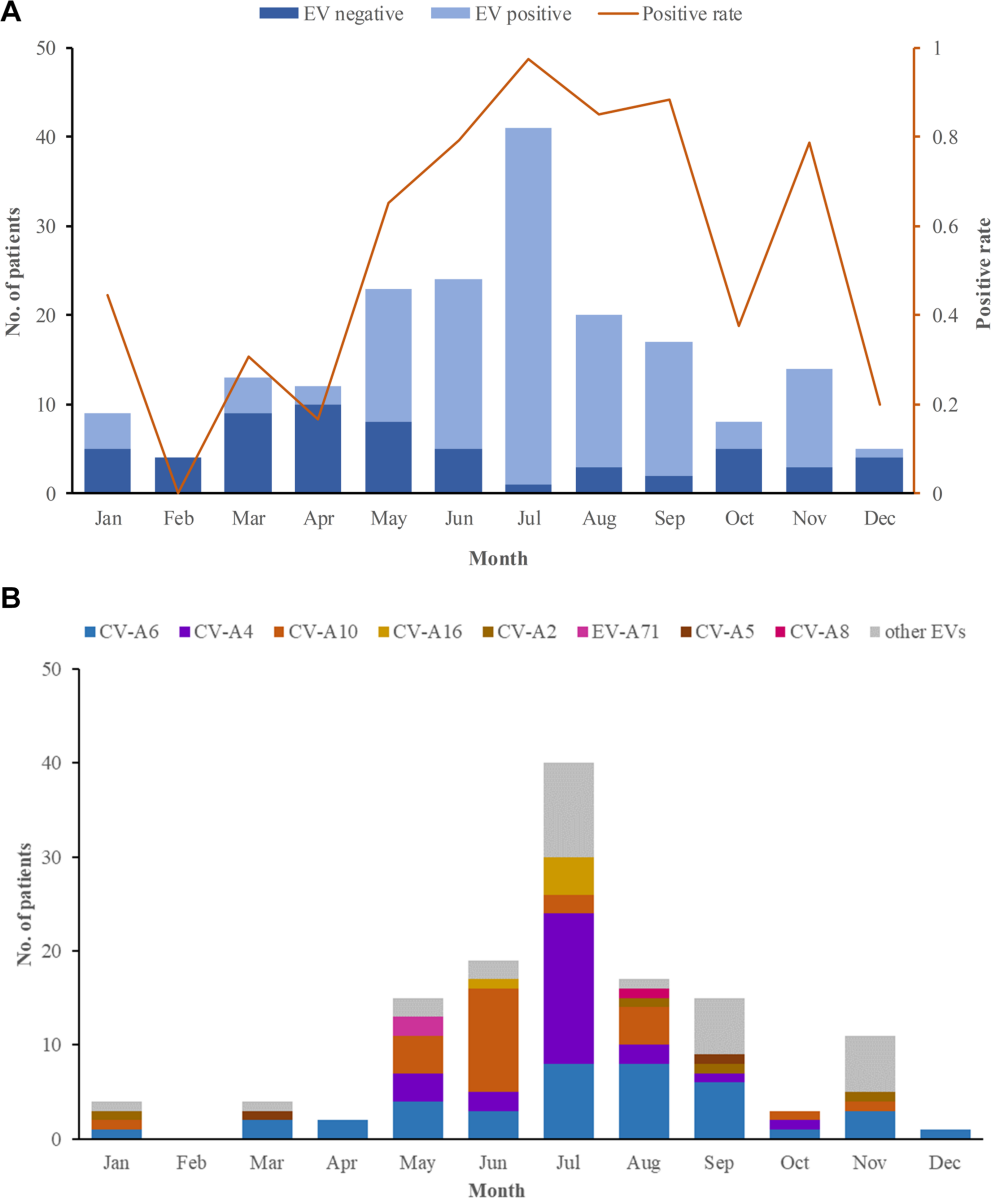
The monthly detection rate of enterovirus (EV) in herpangina children in Tongzhou. (A) The monthly detection rate of EV positive and EV negative samples. (B) The monthly detection rate of EV serotypes detected in this study.

In 2018, 184 herpangina children were monitored by CDC in Tongzhou routinely, and two outbreaks involved 6 children were reported. Among the 190 herpangina children enrolled in this study in 2018, the median age of was 4.2 (IQR: 2.8–5.5) years old and 99 (52.1%) were boys. The percentages of children aged 0–3 years old, 3–5 years old, and ≥5 years old were 26.3%, 43.7% and 30.0%, respectively (Table 1). The predominant epidemic peak of herpangina children was in the summer season (from May to August) (Fig. 1A).

**Table 1:** The demographics characteristics and clinical manifestations of the herpangina children.

Characteristics	Total (*N* = 190)	CV-A4 (*n* = 25)	CV-A6 (*n* = 39)	CV-A10 (*n* = 24)	*P* value
Age, years, Median (IQR)	4 (3–5)	4 (4–5)	5 (4–6)	4 (3–8)	0.427
0–3 years, n (%)	50 (26.3)	4 (2.1)	5 (2.6)	6 (3.1)	0.566
3–5 years, n (%)	83 (43.7)	15 (7.9)	19 (10.0)	11 (5.8)	
≥5 years, n (%)	57 (30.0)	6 (3.2)	15 (7.9)	7 (3.7)	
Sex, male, n (%)	99 (52.1)	9 (36.0)	26 (66.7)	13 (54.2)	0.056
Fever, n (%)	181 (95.3)	25 (100)	37 (94.9)	24 (100)	0.276
Herpes, n (%)	181 (95.3)	22 (88.0)	38 (97.4)	20 (83.3)	0.140
Rhinorrhea, n (%)	1 (0.5)	0 (0)	0 (0)	0 (0)	–
Pneumonia, n (%)	1 (0.5)	0 (0)	0 (0)	0 (0)	–
Encephalitis, n (%)	1 (0.5)	0 (0)	0 (0)	0 (0)	–

### Enterovirus serotypes

Among the 190 herpangina children, 131 were detected to be positive for enteroviruses with a detection rate of 69.0%. Among the 131 children infected with EVs, eight serotypes were identified in this study, including CV-A6 (39/131, 20.5%), CV-A4 (25/131, 19.1%), CV-A10 (24/131, 18.3%), CV-A16 (5/131, 3.8%), CV-A2 (4/131, 3.1%), EV-A71(2/131, 1.5%), CV-A5 (2/131, 1.5%) and CV-A8 (1/131, 0.8%), while 29 had unidentified serotypes. The predominant epidemic peak of CV-A6 was July and August. The peak of CV-A4 was in July and the peak of CV-A10 was in June, which was described in Fig. 1B.

### Clinical manifestation and laboratory results

**Figure 2:**
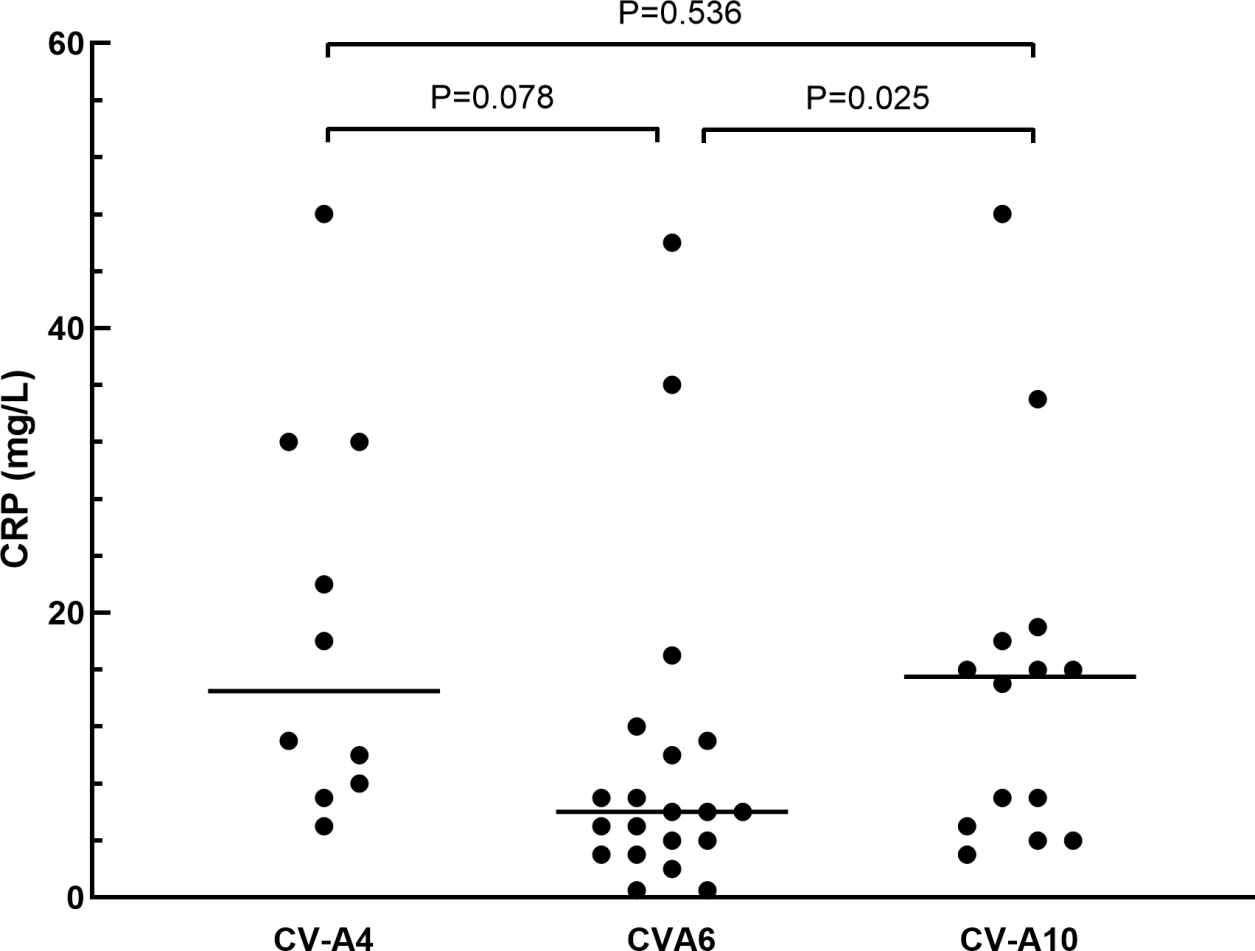
The comparison of C-reactive protein in herpangina children with serotypes coxsackievirus A4 (CV-A4), coxsackievirus A6 (CV-A6) and coxsackievirus A10 (CV-A10). The horizontal line in the scatter plot indicated the median of the set of values. The age and gender of patients in each group were adjusted for the comparison, and *p* = 0.05 was set as the cut-off value to distinguish whether the comparison was statistically significant or not.

The clinical manifestations of herpangina children are shown in Tables 1 and 2. Most of the cases (99.5%) were mild, except for one patient with non-EVs infection who had encephalitis and pneumonia. Fever was developed in 95.3% (181/190) of the herpangina children, and 95.3% (181/190) demonstrated herpes. We compared the clinical manifestations of herpangina children infected with three prevalent EVs (CV-A6, CV-A4 and CV-A10) and no significant differences were found between these among groups. As for the laboratory test results of herpangina children, 68.2% (73/107) children showed high white blood cells (>10 ×10^9^/L), 51.4% (55/107) high percentage of neutrophils (>70.0%), and 48.6% (52/107) low percentage of lymphocytes (<20.0%). An elevated serum C-reactive protein (CRP) level was noted among group and the CRP levels in children infected with CV-A10 and CV-A4 were higher than in those infected with CV-A6 adjusted for age and sex (*P* = 0.025 and *P* = 0.078, Fig. 2).

**Table 2:** The laboratory test results of the herpangina children.

Characteristics	Total (*n* = 107)	CV-A4 (*n* = 10)	CV-A6 (*n* = 20)	CV-A10 (*n* = 14)	*P* value
WBC ×10^9^/L, Median (IQR)	12(9.6–15.9)	12.9(10.5–15.5)	11.3(9.9–14.7)	13.5(9.8-17)	0.646
WBC >10 ×10^9^/L, n (%)	73(68.2)	8(80.0)	14(70.0)	10(71.4)	0.796
Neutrophils%, Median (IQR)	71.2(57.1–79.4)	69.8(60.1–73.6)	74.5(63.9–80.6)	73.3(66.7–80.4)	0.529
>70.0%, n (%)	55(51.4)	5(50.0)	13(65.0)	9(64.3)	0.479
<50.0%, n (%)	22(20.6)	1(10.0)	3(15.0)	0(0)	
Lymphocyte%, Median (IQR)	20.4(12.7–31.8)	19.4(17.1–29.3)	14.2(10.7–26.7)	16.7(12.5–21.9)	0.636
>40.0%, n (%)	20(27.8)	1(14.3)	2(14.3)	0(0)	0.759
<20.0%, n (%)	52(48.6)	6(60.0)	12(60.0)	8(57.1)	
Monocyte%, Median (IQR)	7.7(6.1–10)	9(8–10.8)	7(5.7–9.5)	8(6.5–11)	0.295
>8.0%, n (%)	48(44.9)	7(70.0)	7(35.0)	7(50.0)	0.309
PLT ×10^9^/L, Median (IQR)	247(212–306)	248.5(172–291)	223(190.5-288)	242(189–279)	0.999
>300 ×10^9^/L, n (%)	30(28.0)	2(20.0)	5(25.0)	2(14.3)	0.458
MPV, fL, Mean (SD)	9.8(0.9)	10.0(0.6)	10.0(0.9)	10.0(0.9)	0.307
>10.0 fL, n (%)	45(42.1)	5(50.0)	11(55.0)	7(50.0)	0.346
CRP, mg/L, Median (IQR)	9.0(4–17)	14.5(8–32)	6(3.5–10.5)	15.5(5–18)	0.025^a^
>8 mg/L, n (%)	54(50.5)	7(70.0)	6(30.0)	8(57.1)	0.082

**Notes.**

a The difference among groups was statistically significant, *P* < 0.05.

### Phylogenetic analysis

In laboratory-confirmed cases of enteroviruses, 22 sequences of CV-A4, 36 sequences of CV-A6 and 24 sequences of CV-A10 were amplified by using specific primers in the VP1 region. Based on comparisons with all available VP1 gene sequences downloaded from GenBank, the global phylogenetic analysis of CV-A4, CV-A6 and CV-A10 was performed.

**Figure 3:**
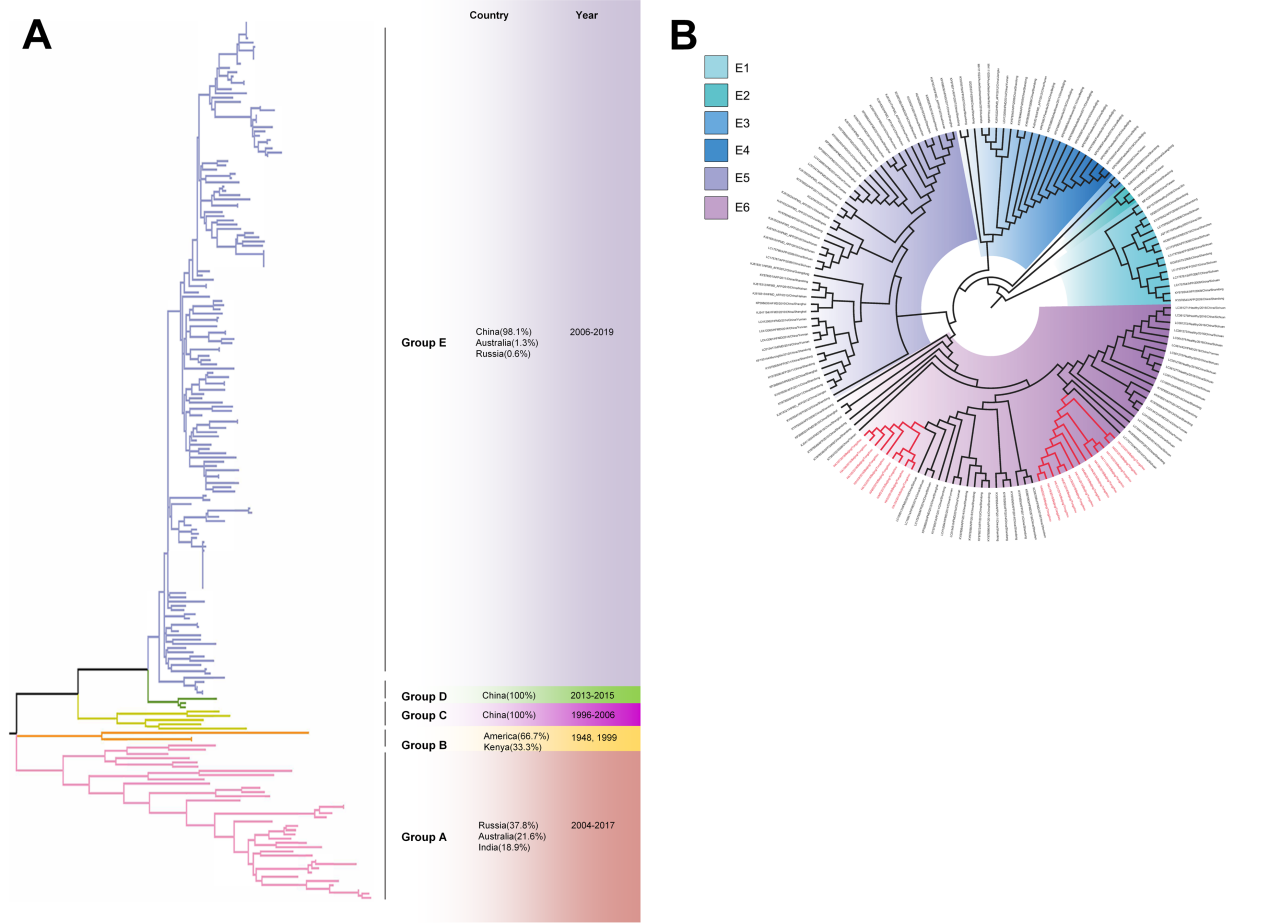
The phylogenetic tree of coxsackievirus A4 (CV-A4) strains based on VP1 genes. Different branch colors represented different subgroups, and the red branches were those Tongzhou strains detected this research. (A) Phylogenetic tree of all CV-A4 strains; All sequences were divided into five groups (Group A–E) according to the structure of the tree. The top three countries in which the subgroup strains were mainly distributed were listed. The year showed temporal dimension of strains in this subgroup. (B) Phylogenetic tree of CV-A4 strains in Group E. Group E was further divided into six subgroups.

**Figure 4:**
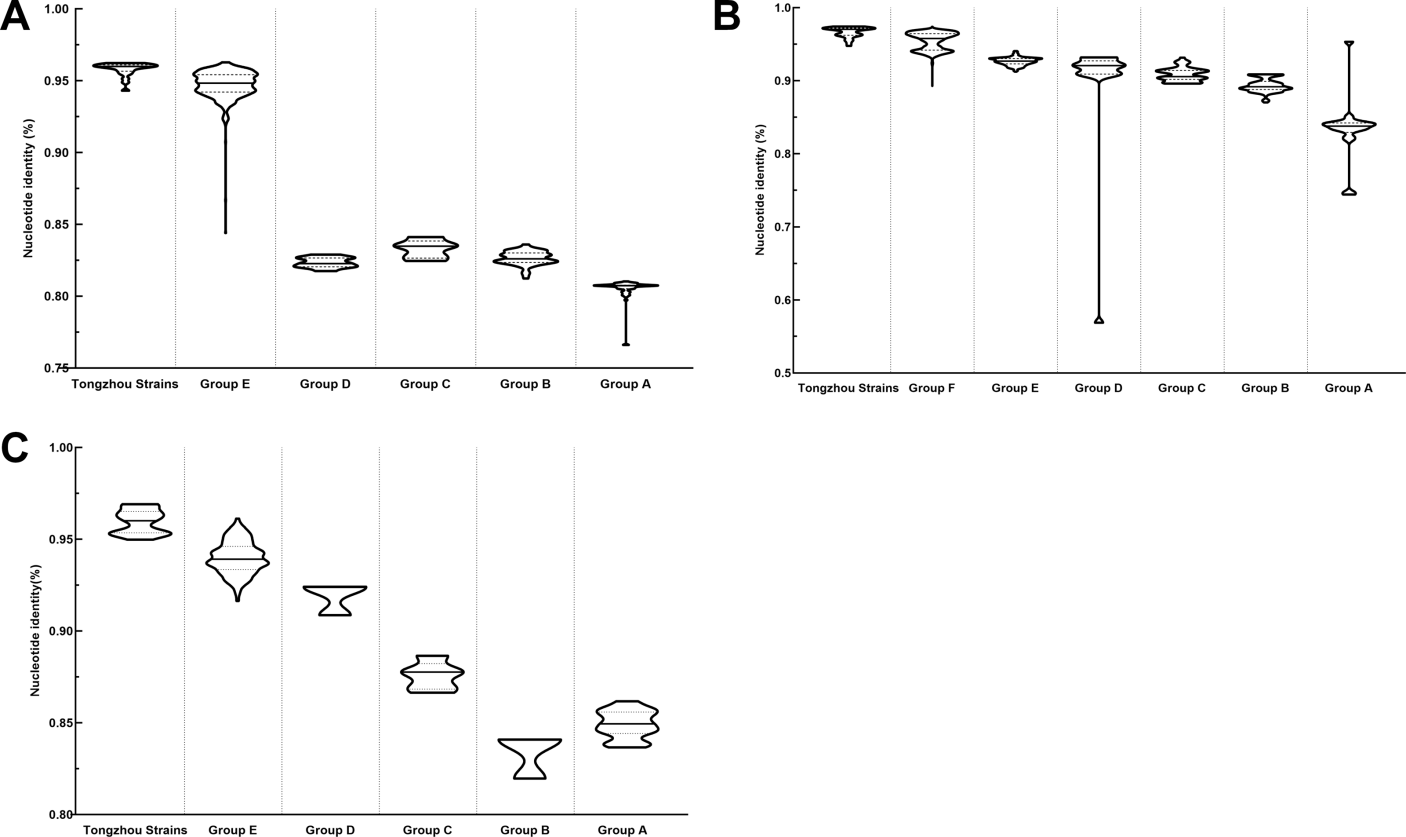
Nucleotide identities of Tongzhou strains with intergroup and intragroup on the basis of VP1 gene. Each violin graph represented the nucleotide identity between different groups with Tongzhou strains. The solid line indicated the median homology, and the dashed line indicated the quartile. (A) intragroup and intergroup nucleotide identities of coxsackievirus A4 (CV-A4) strains; (B) intragroup and intergroup nucleotide identities of coxsackievirus A6 (CV-A6) strains; (C) intragroup and intergroup nucleotide identities of coxsackievirus A10 (CV-A10) strains.

The phylogenetic tree of CV-A4 was constructed with 186 VP1 sequences and 22 amplified Tongzhou strains, and five groups were clustered (Group A-E) in Fig. 3A. CV-A4 strains from Tongzhou showed 84.9%, 83.4%, 87.6%, 91.9% and 94.0% average nucleotide identities with Group A, Group B, Group C, Group D and Group E, and 96.0% homologies intragroup (Fig. 4A). Group A covered strains circulating globally, which indicated that Group A spread and co-evolved across countries. However, almost all the Chinese strains were concentrated in Group E (94.5%), suggesting that this group has become the predominant serotype responsible for the epidemic of CV-A4 in China. Group E was further divided into six additional subgroups (E1–E6) as shown in Fig. 3B. Among them, all Tongzhou CV-A4 sequences were classified in Group E6, which were close to Shandong strains (2011–2014) and Sichuan strains (2014–2015) of China.

**Figure 5:**
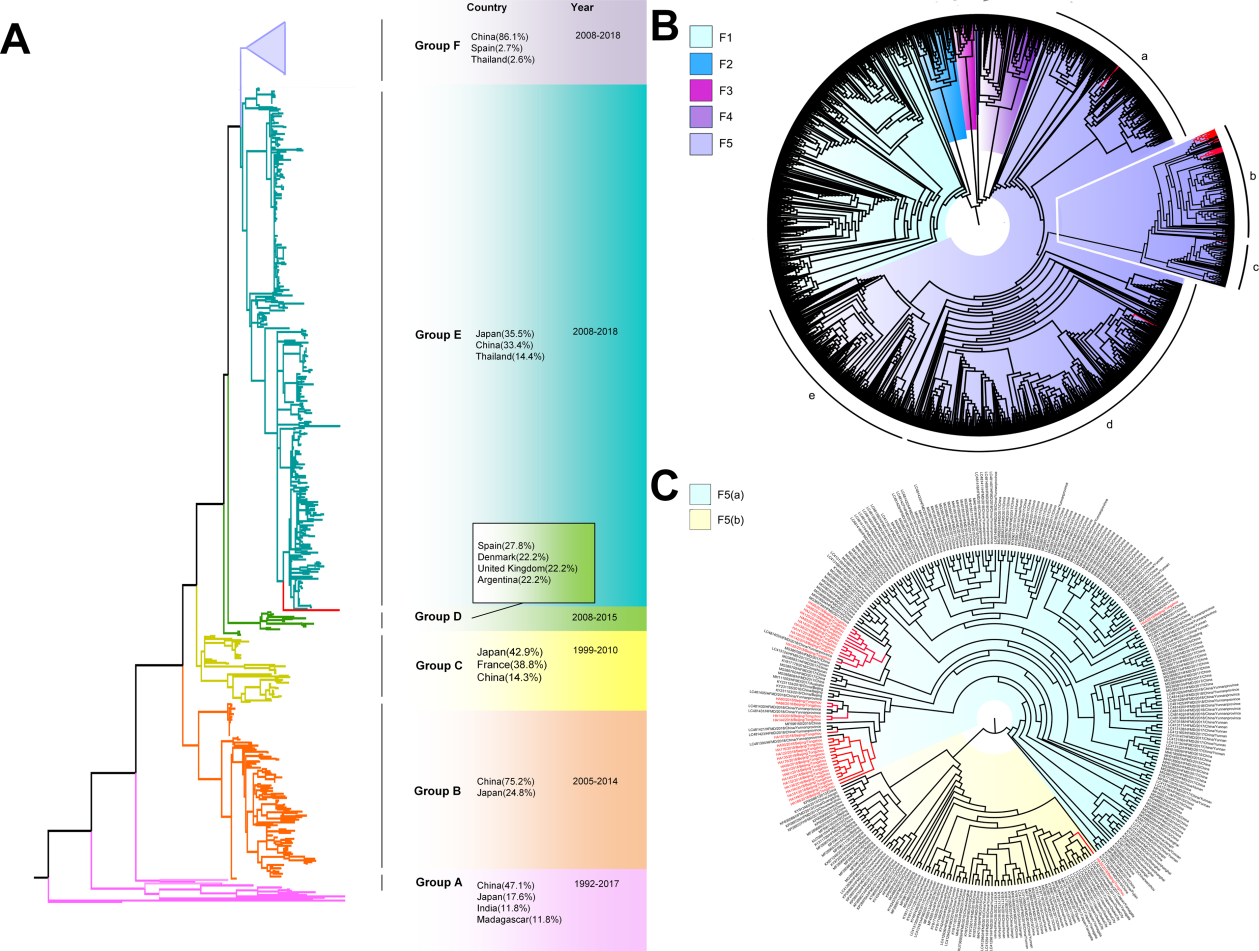
The phylogenetic tree of coxsackievirus A6 (CV-A6) strains based on VP1 genes. Different branch colors represented different subgroups, and the red branches were the Tongzhou strains detected this research. (A) Phylogenetic tree of all CV-A6 strains; All sequences were divided into six groups (Group A–F) according to the structure of the tree. The top three countries in which the subgroup strains were mainly distributed were listed. The year showed temporal dimension of strains in this subgroup. (B) Phylogenetic tree of CV-A6 strains in Group F. (C) Phylogenetic tree of CV-A6 strains in Group F5(b) and Group F5(c).

The phylogenetic tree of CV-A6 was constructed using 3470 VP1 sequences. All sequences were clustered into six groups (Group A-F). For CV-A6, 83.3%, 89.4%, 90.9%, 90.0%, 92.6% and 95.4% average nucleotide identities were calculated between Tongzhou strains with Group A, Group B, Group C, Group D, Group E and Group F, and the homology of Tongzhou strains intragroup was 96.5% (Fig. 4B). A majority of Tongzhou CV-A6 strains (35/36) detected in this study were distributed in Group F, which was mostly made up of Chinese strains (86.1%), Spain strains (2.75%) and Thailand strains (2.6%). One amplified strain was located in Group E, mainly including the strains from Fukuoka (2015-2016), Yamagata (2015) and Osaka (2015) of Japan (Fig. 5A). It was assumed that this strain might have been imported from Japan. Group F was divided into five subgroups (F1-F5), and the residual 35 strains of Tongzhou were distributed in Group F5 entirely (Fig. 5B). We further segmented Group F5 into five additional subgroups (F5a-F5e). Among them, one strain belonged to Group F5a, which was intimately related to the Yunnan, Shanghai and Jiangxi strains previously reported in 2015–2017 of China. One strain belonged to Group F5d, which was close to the Chinese strains from 2015 to 2017 (Fig. 5B). The remaining 33 strains were entirely located on two branches (F5b and F5c), as shown in Fig. 5C. Subgroup F5b was composed of 195 Chinese strains in 2012–2018, and one Australian strain in 2017, while subgroup F5c mainly consisted of Chinese strains from 2013 to 2017 and Japanese strains in 2017. The circulation of CV-A6 had a distinct spatiotemporal cluster. We observed that the predominant serotypes were Group B with Group C co-circulating before 2008, but after 2008 the dominating ones were Group E and Group F. Almost all the CV-A6 strains circulating contemporarily were in Group F. Furthermore, Group F1 strains were circulated mainly during 2008–2012, but later, Group F5 strains were the primary epidemic (Fig. S1B).

**Figure 6:**
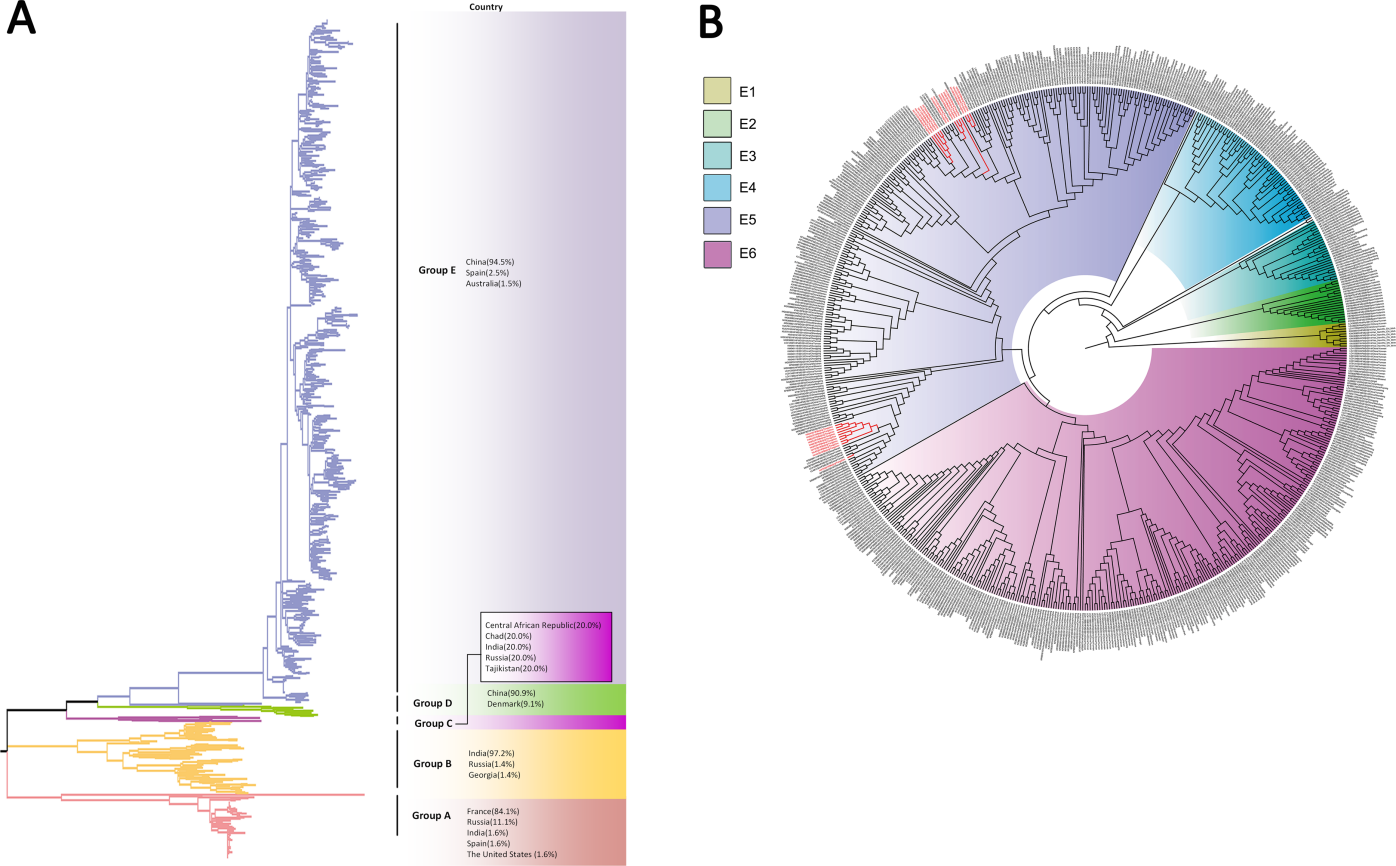
The phylogenetic tree of coxsackievirus A10 (CV-A10) strains based on VP1 genes. Different branch colors represented different subgroups, and the red branches were those Tongzhou strains detected this research. (A) Phylogenetic tree of all CV-A10 strains; All sequences were divided into five groups (Group A–E) according to the structure of the tree. The top three countries in which the subgroup strains were mainly distributed were listed. The year showed temporal dimension of strains in this subgroup. (B) Phylogenetic tree of CV-A10 strains in Group E. Group E was further divided into six subgroups.

Based on VP1 gene sequences of CV-A10, 801 VP1 sequences and 24 amplified sequences in this study were constructed for the phylogenetic tree. All the strains were classified into five groups (Group A-E). We analyzed the sources of different branches and found that 98.1% of the sequences of Group E were obtained from China (Fig. 6A). CV-A10 strains from Tongzhou revealed 80.5%, 82.6%, 83.3%, 82.3% and 94.7% average nucleotide identities with Group A, Group B, Group C, Group D and Group E, and 95.8% homologies intragroup (Fig. 4C). The phylogenetic tree showed that Group E was divided into six additional subgroups (E1-E6) in Fig. 6B. All the amplified CV-A10 strains were distributed in two groups in Group E5. Ten Tongzhou strains were clustered in a group which was consist of Beijing strains, Henan strains, Yunnan strains and Guangdong strains in 2016–2018, while the remaining 14 Tongzhou stains were largely close to Yunnan strains (2017–2018) in China. Spatiotemporal characteristics were discovered in the subgroups of Group E, which showed that the ascendant cluster was Group E6 between 2009 and 2013. But it was converted to Group E5 after 2013 (Fig. S1C).

## Discussion

There have been a number of outbreaks of herpangina in various regions of the world, most of which were dominated by CV-A2 and CV-A10 strains. CV-A2 infection was prevalent in Thailand (2015) (Chansaenroj et al., 2017), Chinese Taiwan (2008) (Lee et al., 2011), Guangdong (2015) (Peng et al., 2016) and Zhejiang Province (2015)(Li et al., 2016c), while CV-A10 infection was prevalent in France (2010) (Mirand et al., 2012), and Tianjin City (2014-2016) (Li et al., 2018a; Li et al., 2016a) of China. In addition, there are also outbreaks of CV-A5, CV-A16 and CV-A4 (Huo et al., 2017; Park et al., 2012; Shima et al., 2007). However, there have been few reports on CV-A6 outbreaks, except for studies in Jilin Province and Guangdong Province of China with a small sample size, possibly because no large-scale outbreak has ever occurred (Shuang et al., 2016; Wang et al., 2016; Zeng et al., 2017). In this study, we found that the preponderant pathogen of herpangina was CV-A6, followed by CV-A10 and CV-A4 in Beijing, during 2018, which may indicate that the pathogen spectrum of herpangina was changing to CV-A6. Among the positive samples, 29 enterovirus positive samples were not further sequenced and serotyped due to the limited sequencing conditions and low virus concentration. This result suggested that the epidemiological monitoring of herpangina and variations in its pathogen spectrum were worthy of further investigation. It is worth noting that HFMD also has undergone a similar spectrum shift. As an emerging pathogen, CV-A6 has gradually replaced EV-A71 and CV-A16 to become the mainstream serotype of HFMD since 2013 (Li et al., 2018b; Lianlian et al., 2015). Meanwhile, it should be noted that the predominant pathogen of herpangina was more variable. Thus, more monitoring data is required to ascertain molecular epidemiology of herpangina (Shima et al., 2007).

CV-A6 strains were clustered into six groups, and CV-A4 and CV-A10 were clustered into five groups based on the VP1 gene sequences due to the large number of viral strains, as in other studies (Bian et al., 2019; Chen et al., 2018; Ji et al., 2019; Song et al., 2017). Our CV-A6 strains were primarily distributed in Group F5, and one strain located in Group E was imported from Japan. All the amplified CV-A4 and CV-A10 strains were located in Group E. The groups of coxsackievirus were highly dynamic, but all the strains in our study were close to those isolated from other cities in China and neighboring counties, which indicated that geographic factors may contribute much to the prevalence of herpangina. In addition, coxsackievirus also exhibited different genetic characteristics regarding their temporal variations. We also found that herpangina strains could not be differentiated from HFMD stains based on the phylogenetic analysis. This was analogous to Park K’s conclusion that no significant differences were found between HFMD and herpangina on the basis of VP1 gene sequences (Park et al., 2012). Further studies are required to provide more evidence relating to the phylogenetic and spatiotemporal pattern of herpangina.

## Conclusions

CV-A6 was the predominant pathogen in children with herpangina in Tongzhou, followed by CV-A4 and CV-A10, which implied that CV-A6 was emerging as another predominant serotype of herpangina. The circulation of CV-A6 and CV-A10 had spatiotemporal cluster. Meanwhile, the level of CRP may have implications for differentiating EV infection. In controlling the transmission of herpangina, the surveillance and reporting system should be enhanced. In controlling the transmission of herpangina, the surveillance and reporting system should be enhanced. New vaccines against multiple coxsackieviruses, especially CV-A6, should be developed as soon as possible. It is also important to increase the classification of EVs in children with herpangina for the further surveillance and prevention of communicable diseases.

##  Supplemental Information

10.7717/peerj.9991/supp-1 Supplemental Information 1 Primers for amplification and sequencing of EVs,

10.7717/peerj.9991/supp-2 Supplemental Information 2 The details of included strains acquired from GenBank

10.7717/peerj.9991/supp-3 Supplemental Information 3 Analysis of temporal distribution characteristics based on VP1 gene sequences of coxsackievirus A4 (CV-A4), coxsackievirus A6 (CV-A6) and coxsackievirus A10 (CV-A10) The shade of color in the figure represented the number of detected sequences for the group in that year. The dark color indicated a large number of sequences, and the light color represented a low number of sequences. A: The temporal distribution of CV-A6; B: The temporal distribution of CV-A4; C: The temporal distribution of CV-A10.

10.7717/peerj.9991/supp-4 Supplemental Information 4 Raw data All demographic characteristics, clinical manifestation and laboratory indicators of herpangina children.

10.7717/peerj.9991/supp-5 Supplemental Information 5 Sequence data

## Additional Information and Declarations

### DNA Deposition

The following information was supplied regarding the deposition of DNA sequences: The group CV-A4 sequences are available at GenBank: MN864893–MN864917. The group CV-A6 sequences are available at GenBank: MN864918–MN864953. The group CV-A10 sequences are available at GenBank: MN864954–MN864977.

### Data Availability

The following information was supplied regarding data availability: The raw measurements are available in the Supplementary Files.
