# Supplementary material for: Molecular epidemiology and clinical characteristics of herpangina children in Beijing, China: a surveillance study
Source: PeerJ. 2020 Oct 15;8:e9991. doi: 10.7717/peerj.9991 (PMC7568857; doi:10.7717/peerj.9991)
Supplement: Supplemental Information 1 [file peerj-08-9991-s001.docx]

Supplemental Table 1. Primers for amplification and sequencing of enteroviruses.

| Genes | Oligo name | Sequence (5’-3’) | Product(bp) | Reaction condition |
| --- | --- | --- | --- | --- |
| HRV/HEV 5’NTR | 164F | CAAGCACTTCTGTTTCCC | 337 bp | \| Denaturalization: 95℃ 3min; Annealing: 50℃ 30min; Amplification: (95℃ 30s, 55℃ 30s, 72℃ 30s) for 38 cycles with a final extension at 72℃ 7min. \| \| --- \| \| |
|  | 501R | CACGGACACCCAAAGTAGT |  |  |
| HRV-C VP4/2 | HRV-F | GGGACCAACTACTTTGGGTGTCCGTGT | 549 bp | \| Annealing: 50℃ 30min; Denaturalization: 95℃ 3min; Amplification: (95℃ 30s, 60℃ 45s, 72℃ 45s) for 38 cycle with a final extension at 72℃ 7min \| \| --- \| \| |
|  | HRV-R | GCATCIGGYARYTTCCACCACCANCC |  |  |
| CVA6-VP1 | CVA6-VP1-F | CTTCGTAGTGCCACCAGATA | 1090 bp | \| Annealing: 50℃ 30min; Denaturalization: 95℃ 3min; Amplification: (95℃ 30s, 51℃ 30s, 72℃ 1min 10s) for 38 cycle with a final extension at 72℃ 7min \| \| --- \| \| |
|  | CVA6-VP1-R | GTGGCGAGATGTCGGTTTA |  |  |
| CVA4-VP1 | VP1A (2319) | CTTCGTAGTGCCACCAGACAC | 1066 bp | \| Annealing: 50℃ 30min; Denaturalization: 95℃ 3min; Amplification: (95℃ 30s, 51℃ 30s, 72℃ 1min 10s) for 38 cycle with a final extension at 72℃ 7min \| \| --- \| \| |
|  | VP1S (3384) | AGCTCCAGATTGTTGACCGA |  |  |
| CVA10-VP1 | CV-A10 VP1F | TGACCCTGTGGAGGATAT | 800 bp | \| Annealing: 50℃ 30min; Denaturalization: 95℃ 3min; Amplification: (95℃ 30s, 53℃ 30s, 72℃ 1min) for 38 cycle with a final extension at 72℃ 7min \| \| --- \| \| |
|  | CV-A10 VP1R | CATATTAGCTTGCTTGATGC |  |  |
