# Supplementary material for: Molecular epidemiology and clinical characteristics of herpangina children in Beijing, China: a surveillance study
Source: PeerJ. 2020 Oct 15;8:e9991. doi: 10.7717/peerj.9991 (PMC7568857; doi:10.7717/peerj.9991)
Supplement: Supplemental Information 2 [file peerj-08-9991-s002.docx]

**Supplemental Table 2. The details of included strains acquired from GenBank**

| **CV-A4 Strains** | | | | |
| --- | --- | --- | --- | --- |
| LC481442 | KY978549 | KJ818315 | GQ253375 | KR185978 |
| LC361271 | KJ541163 | KC867061 | MF422546 | KC879492 |
| LC361270 | KP398832 | KP398834 | JQ715709 | LT719044 |
| LC361272 | KY978543 | KC867062 | GQ253372 | KC879506 |
| LC361273 | KY978547 | KJ818320 | KY978542 | KC879503 |
| LC361274 | KJ818321 | KJ818317 | LC175760 | KC879547 |
| LC361275 | KY978550 | KC867065 | JQ715710 | AY919423 |
| LC361278 | KY978558 | KU380270 | KC867060 | MH111024 |
| LC361277 | KP398840 | KU380269 | LC175765 | MH111025 |
| LC361276 | KY978556 | KX982676 | LC175759 | MH111026 |
| LC169515 | KY978555 | KJ818319 | GQ253373 | MH111022 |
| KY978563 | KF150144 | KP398836 | LC175763 | JN203510 |
| KY978561 | LC013417 | KY978571 | LC175761 | KC879531 |
| KY978569 | LC412061 | KY978570 | LC175764 | KC879525 |
| LC013472 | LC412063 | GQ253374 | KY978544 | KC879530 |
| LC412065 | LC412062 | MH111023 | KY978540 | KC879512 |
| LC175768 | KJ541164 | MH111029 | LC412060 | KC879520 |
| LC169513 | KP398835 | KJ818322 | KJ818307 | KC879519 |
| KY978559 | KJ818313 | LC412059 | KJ818306 | JN203511 |
| LC175770 | KJ818312 | KY978545 | KJ818309 | KC879546 |
| KC867064 | KY978551 | KY978546 | KJ818308 | JN203507 |
| KC867063 | KJ818311 | KY978539 | LC169516 | JN203513 |
| KY978553 | LC175767 | KJ818318 | KP289442 | JN203505 |
| KY978565 | LC175766 | KP676957 | KY978560 | JN203506 |
| KY978562 | KJ818316 | KP676985 | GQ253376 | JN203509 |
| KY978554 | KJ818314 | KP676962 | KY978567 | MH111020 |
| KY978566 | KJ818325 | KP676986 | KY978541 | KY271949 |
| KY978572 | KY978548 | KP676984 | GQ253377 | MH111021 |
| KY978568 | KJ818323 | KP676958 | JQ715708 | MH111027 |
| KY978564 | KJ818324 | KP676965 | GQ176232 | MH111028 |
| LC013451 | KC879539 | KP676964 | AY421762 | KY978538 |
| LC412064 | KY978552 | KP676963 | AF081295 | HQ728260 |
| KY978557 | KJ818305 | KP676961 | KR185979 | MF422545 |
| KP398839 | LC013473 | KP676960 | KC879538 | MG550920 |
| LC175769 | LC412066 | KP676959 | KC879498 | LC175758 |
| LC169514 | KP398837 | MF422544 | KC879555 | KJ818310 |
| LC169517 | KP398833 | KY978537 | LC175762 | KP398838 |
| KT353722 |  |  |  |  |

| **CV-A6 Sequences** | | | | |
| --- | --- | --- | --- | --- |
| LC421650 | KJ848301 | KM079540 | KY856716 | MF596100 |
| LR027552 | MF991295 | KM079531 | LC413166 | MF285647 |
| KF412903 | MF962682 | KM079532 | LC413139 | MF596093 |
| LT719048 | KT779412 | KM079526 | MG385776 | MF285640 |
| LT719047 | MH544984 | KM079519 | MG385754 | MF596106 |
| KC866915 | MH544982 | KM079578 | MG385751 | MF285652 |
| KP143073 | MH544970 | KM079522 | MH018523 | MF596090 |
| KP143074 | KY424411 | MF373605 | MH018522 | MF596089 |
| KP143078 | KU958491 | KJ865444 | MH018521 | MF285637 |
| LC421656 | KP289620 | KJ865447 | MG385820 | MF285636 |
| LC421592 | KP289392 | KF836579 | LC481418 | KP289479 |
| KP143076 | KP289389 | KT985015 | LC481417 | KY972305 |
| KP143075 | KP289702 | KT985014 | LC481401 | KX871240 |
| JQ364886 | KP289974 | KY424420 | LC481400 | KF836567 |
| AY421764 | KP289991 | KJ577315 | LC481395 | KY424398 |
| AF081297 | KP289742 | KJ577340 | LC481392 | KF647880 |
| JQ364887 | KP289470 | KC414743 | LC413161 | KY913464 |
| JN203517 | KP289460 | KC207832 | MH018514 | KP289895 |
| KP143079 | KP289936 | KY913440 | MG385815 | KP289865 |
| MF596069 | KP289682 | KY913439 | MG385797 | KP289838 |
| MF285625 | KP289938 | KY913436 | LC481428 | KP289817 |
| MF596067 | KP289520 | KJ577312 | LC481427 | KP289851 |
| MF285623 | KP289587 | KJ577283 | LC481422 | KP289833 |
| JX495127 | KP289510 | KJ577281 | MH018513 | KP289706 |
| JX495126 | KP289807 | KJ577293 | MH018512 | KP289818 |
| KJ609188 | KP289615 | KJ577280 | LC481433 | KP289771 |
| KJ577289 | MH544981 | KJ577301 | MG385798 | KP289825 |
| KY913429 | MF596103 | JX473334 | MH018515 | KP289604 |
| JX473340 | MF285649 | KJ577292 | MG385793 | KP289472 |
| KY424419 | KT985023 | KJ577300 | MG385764 | KP290000 |
| KT779410 | KY211701 | JX495137 | MG385801 | KP289849 |
| KM279379 | KT985019 | KJ156350 | MG385795 | KP289842 |
| KY424406 | KX064298 | KM079558 | MH018518 | KP289906 |
| KY424374 | KU212263 | KJ865424 | MH018520 | KP289989 |
| KJ743209 | KX064288 | JX154929 | MH018519 | KP289956 |
| KJ577273 | KU736934 | KY913434 | MG385826 | KP289967 |
| KY424408 | KX064287 | KJ743212 | MH018517 | KP289900 |
| KT985026 | KU736935 | JX473343 | MH018516 | KP289796 |
| KT985011 | KJ577389 | KJ577290 | LC481416 | KP289599 |
| KT124610 | MF991297 | JX495134 | LC481411 | KP289987 |
| KT124607 | MF991296 | KX189190 | LC481408 | KP289902 |
| KJ848296 | MF962687 | KF836601 | LC481407 | KP289898 |
| KT124601 | MF962686 | JX473335 | LC481406 | KP289812 |
| KY424390 | MF962685 | KY913433 | MG385825 | KP289717 |
| KY424358 | MF962684 | KJ865429 | MG385823 | KP289651 |
| KT985012 | MF962683 | KM079577 | MG385767 | KP289650 |
| KT985010 | KJ541390 | KM079557 | MG385784 | KP289649 |
| KJ577387 | KJ541371 | KM079559 | KY856715 | KP289648 |
| KU708572 | KJ541400 | KM079504 | KY856712 | KP289617 |
| MF596068 | KF647884 | KY913430 | MG385829 | KP289606 |
| MF285624 | KU212279 | KM079516 | MG385828 | KP289534 |
| KT124604 | MF596092 | KM079507 | MG385827 | KP289385 |
| KT124602 | MF285639 | KC866904 | MG385774 | KP289819 |
| KY211727 | KY424381 | KM079511 | MG385772 | KP289875 |
| KY424393 | KU212262 | KM079506 | MG385814 | KP289816 |
| JQ964234 | KX064301 | KM079509 | MG385788 | KP289724 |
| JN797598 | KX064306 | KM079508 | MG385757 | KP289951 |
| JX154921 | KU212272 | KC866909 | LC481415 | KP289457 |
| KC866913 | KX064300 | KC866908 | MG385786 | KP289444 |
| KU708574 | KU212268 | KC866918 | MG385782 | KP289443 |
| KJ848297 | KJ541411 | KC866911 | MH018544 | KP289691 |
| KY211690 | KJ541412 | KC866905 | MH018541 | KP289446 |
| JX495129 | KJ541405 | HE572938 | MH018543 | KP289908 |
| KT985016 | MG488226 | HE572928 | MH018542 | KP289876 |
| KT124603 | KY913486 | HE572926 | MH018540 | KY913479 |
| JQ364889 | KY424395 | HE572924 | MH018539 | KY817159 |
| KJ577299 | KJ612513 | HE572922 | LC481396 | KY424386 |
| JX495145 | KJ541381 | HE572918 | MH018532 | MH716167 |
| KU708571 | KJ541160 | HE572915 | MH018531 | MF596107 |
| KU708569 | KX064304 | HE572936 | MH018538 | KY497432 |
| KY211723 | KU212274 | HE572920 | MH018537 | KY913480 |
| KJ577288 | KU212269 | KC866912 | MH018536 | KF647881 |
| KJ577287 | KP289657 | KC866901 | MH018535 | KP289694 |
| MF596066 | KJ541396 | KM079510 | MH018534 | KP289685 |
| MF285622 | KY424426 | KJ865422 | MH018533 | KJ743232 |
| JX495118 | KP005922 | KX372340 | MH018530 | LC013425 |
| KC866917 | KP005921 | KX212514 | MH018529 | LC412046 |
| KJ743213 | KP005881 | KX212347 | MH018525 | LC013424 |
| KC867008 | KP005916 | KX212518 | MH018524 | KY424401 |
| KJ577297 | KP005896 | KX212467 | MH018528 | KP289891 |
| JX495119 | KM079588 | KX212462 | MH018527 | KP289879 |
| KC866916 | KJ018114 | KX212464 | MH018526 | KP289652 |
| KC866914 | MF962689 | KX212463 | MG385830 | KJ743215 |
| KC866903 | KT124605 | KX212471 | LC413143 | KP289582 |
| JX495130 | KP289847 | KX212470 | MG385802 | LC412976 |
| KY211711 | KP289792 | KX212461 | LC413129 | LC412975 |
| JX495132 | KP289588 | KX212444 | MG385819 | LC412972 |
| KJ865427 | KP289375 | KX212443 | MG385813 | LC412974 |
| KJ609186 | KJ541415 | KX212472 | KY856718 | LC412973 |
| KJ577285 | KJ541414 | KX212517 | MG385799 | LC412971 |
| JN639896 | KF836603 | KX212473 | MG385818 | LC412969 |
| JN639895 | KF647887 | KX212469 | MG385778 | LC412968 |
| JN655886 | KJ541413 | KX212468 | MG385800 | LC412970 |
| KJ577284 | MF596073 | KX212446 | MG385796 | LC412967 |
| KJ577282 | KJ541383 | KX212516 | MG385824 | MG385803 |
| KC866921 | KJ541162 | KX212459 | KY856709 | LC413148 |
| KC866906 | KF647889 | KX212457 | MG385791 | LC413151 |
| KC866902 | KJ541394 | KX212449 | LC481419 | LC413144 |
| KC866919 | KY913474 | KX212465 | LC481398 | LC413142 |
| JQ364888 | KJ541423 | KX212456 | LC413160 | MF596143 |
| KP143080 | MF991293 | KX212455 | LC413155 | MF285680 |
| KP143077 | MF962678 | KX212450 | MH536772 | LC413140 |
| KC866900 | KJ541398 | KX212454 | MH018511 | LC413133 |
| KM079503 | KF647875 | KX212466 | MG385821 | LC413154 |
| KM079502 | MF962688 | KX212458 | MH018510 | LC413141 |
| JX495141 | MF991292 | KX212447 | MG385787 | LC413137 |
| JX495133 | MF962677 | KX212515 | MG385773 | LC413132 |
| JX495120 | MF596079 | KX212448 | MG385822 | LC413123 |
| MF596065 | MF285628 | KX212453 | MG385817 | LC413136 |
| MF285621 | MF596078 | KX212451 | MG385807 | LC413125 |
| KY211724 | KY211710 | KX212452 | MG385759 | KY856711 |
| KY211707 | MF596075 | KX212445 | MG385806 | MF596109 |
| KP143081 | MF285626 | KU212265 | MG385810 | LC412939 |
| KM079513 | MF962690 | KX212513 | MG385781 | KY424357 |
| KM079512 | KJ848300 | KX212440 | LC481426 | LC412935 |
| AB779617 | KJ541378 | KX212512 | LC481424 | KY211719 |
| LC421628 | KJ541410 | KX212442 | LC481429 | LC412045 |
| LC421629 | KJ541421 | KX212510 | LC481425 | MF596122 |
| LC421627 | KJ541373 | KX212439 | LC481420 | LC430229 |
| LC126149 | KJ541402 | KX212438 | LC481391 | LC413152 |
| LC126147 | KJ541382 | KX212519 | LC481402 | LC413149 |
| LC126148 | KJ541161 | KX212460 | LC481399 | LC413121 |
| LC421626 | KF647888 | KP144341 | LC413158 | LC413157 |
| LC421624 | KT985032 | KP144340 | LC413171 | LC413145 |
| LC421623 | KJ541417 | KX212511 | LC413128 | LC412036 |
| LC421622 | KP289910 | KX212441 | LC413165 | KY817153 |
| LC421618 | KP289860 | LC421638 | LC413147 | LC412032 |
| LC421612 | KP289477 | LC421637 | LC413156 | LC412048 |
| LC421609 | KJ541428 | LC421636 | LC413134 | LC013445 |
| LC421607 | KJ541425 | LC421635 | LC413122 | LC412025 |
| LC421605 | KJ848299 | LC421631 | MH018509 | LC013438 |
| LC421603 | KJ848298 | LC126152 | MH018508 | LC013427 |
| LC421617 | KJ577392 | LC126151 | MH018507 | LC013436 |
| LC421625 | MF596126 | LC421634 | MH018506 | LC412040 |
| LC421621 | MF285666 | LC421633 | MH018505 | LC412027 |
| LC421620 | KY211696 | LC421632 | MG385816 | LC013440 |
| LC421619 | KY211699 | LC421630 | MG385805 | KY211731 |
| LC421616 | MF596140 | KX212351 | MG385752 | LC412052 |
| LC421613 | MF285677 | KX212521 | KY817188 | LC412051 |
| LC421610 | MF596121 | KX212482 | MF596139 | LC013456 |
| LC421604 | MF596102 | MK167116 | LC413180 | LC013455 |
| LC421602 | KY424366 | MK167123 | LC413146 | LC412035 |
| LC421615 | KY913526 | MK167122 | MF455511 | LC412034 |
| LC421608 | KY817165 | MK167113 | MF455510 | LC013448 |
| LC421606 | KY211694 | MK167111 | MG488228 | LC013447 |
| LC421614 | MF596098 | MK167104 | MG488224 | LC412050 |
| LC421611 | MF285645 | MK167088 | KY424356 | LC013454 |
| MF838736 | LC412965 | MK167049 | MG488204 | LC412015 |
| FR797988 | KY211695 | MK167052 | MG488208 | LC013420 |
| HE572939 | KU708606 | MK167110 | MG488207 | LC013435 |
| HE572927 | KJ541401 | MK167108 | MG488206 | LC013421 |
| HE572910 | KP289943 | MK167091 | MG488205 | LC412026 |
| HE572934 | KJ541422 | MK167087 | KX871241 | LC013439 |
| HE572929 | KJ541440 | MK167084 | KY913491 | MH716144 |
| HE572923 | KJ541416 | MK167080 | LC412944 | KY817148 |
| HE572917 | KJ541404 | MK167078 | LC412937 | KY817147 |
| HE572916 | KJ541420 | MK167077 | KY913518 | KY817146 |
| HE572919 | KJ541389 | MK167076 | KY972309 | LC412945 |
| HE572911 | KJ541406 | MK167075 | KU736943 | KY913527 |
| HE572933 | KJ541392 | MK167073 | KX064297 | KY913514 |
| HE572932 | KJ541430 | MK167070 | KU736947 | KY913521 |
| HE572908 | KF647883 | MK167069 | KX871243 | KY913529 |
| HE572931 | KJ541409 | MK167068 | KY913517 | KY913512 |
| HE572921 | KX064302 | MK167065 | KP289888 | KY913511 |
| HE572930 | KU212273 | MK167064 | MF962676 | KY913504 |
| HE572912 | KJ541408 | MK167060 | MF962675 | KY913495 |
| HE572901 | KP289919 | MK167056 | KU708581 | KY913484 |
| HE572935 | KY913478 | MK167074 | KJ743219 | MH018490 |
| MF422554 | KY913461 | MK167071 | KY211705 | MH018488 |
| MF422552 | KY424391 | MK167085 | LC421652 | KU708611 |
| MF422555 | KX189177 | MK167121 | LC421651 | KU708570 |
| MF422553 | KF836555 | MK167120 | LC420006 | MH018489 |
| MF422556 | KM079501 | MK167118 | LC420013 | LC412047 |
| KR706309 | KP005898 | MK167112 | LC420001 | KY211730 |
| JQ946053 | KJ541407 | MK167114 | LC420005 | LC412022 |
| LC421599 | KP005915 | MK167094 | LC420000 | LC013431 |
| LC421595 | KJ577405 | MK167098 | LC419999 | LC013428 |
| LC126145 | KJ541419 | MK167093 | LC421649 | LC412043 |
| AB779614 | KJ541403 | MK167083 | LC421648 | LC412044 |
| LC421601 | KJ541399 | MK167095 | MK167106 | LC412039 |
| LC421593 | KJ541431 | MK167048 | MK167102 | LC412038 |
| LC421600 | KP289597 | MK167119 | MH018484 | LC412037 |
| LC421596 | KU958495 | MK167107 | KY856714 | LC412031 |
| LC421591 | KP289845 | MK167051 | KY856710 | LC013444 |
| LC421590 | KP289844 | MK167047 | MG385775 | LC412016 |
| LC421588 | KP289843 | MK167117 | MG385753 | LC412042 |
| LC421586 | KP289837 | MK167063 | KY817187 | LC412021 |
| LC421587 | KP289761 | MK167053 | KY817175 | LC013429 |
| LC421589 | KP289734 | MK167054 | MG385765 | LC412018 |
| LC421597 | KP289708 | MK167055 | MG385749 | LC013434 |
| LC421594 | KP289640 | MK167058 | MH716170 | LC013423 |
| AB779616 | KP289580 | MK167061 | LC412955 | LC412020 |
| LC421598 | KP289569 | MK167089 | LC412952 | LC412033 |
| LC126146 | KP289503 | MK167066 | KY913513 | LC013446 |
| LC126144 | KP289387 | MK167086 | MH018483 | LC013426 |
| LC126143 | KP289391 | MK167072 | KY913525 | LC412017 |
| FR797987 | KP289492 | MK167099 | KY817182 | LC013422 |
| FR797986 | KP289515 | MK167081 | KY817174 | LC412029 |
| FR797985 | KP289926 | MK167079 | KY817173 | LC013442 |
| FR797984 | KP289836 | MK167062 | KY817172 | LC412030 |
| KX212476 | KP289709 | MK167092 | LC412961 | LC013443 |
| KX212527 | KP289703 | KX212524 | LC412941 | KY972303 |
| KX575864 | KP289855 | KX212477 | LC412940 | KU212275 |
| KX575862 | KP289840 | KX212523 | MH716169 | KU736942 |
| KX575865 | KP289468 | KX212478 | KY972304 | KY424412 |
| KX575863 | KP289935 | KX212522 | KY972301 | KU212271 |
| MK086189 | KP289673 | KX212479 | KY972300 | KY424384 |
| KX212526 | KP289917 | KX212525 | KU736939 | LC412028 |
| KX212475 | KP289880 | KX212480 | MH018482 | LC013441 |
| KX212474 | KP289798 | KP144348 | KY817167 | KY211732 |
| KP144353 | KP289786 | KP144347 | LC412943 | LC412019 |
| KP144351 | KP289942 | KX212520 | LC412936 | KP289380 |
| KP144352 | KP289719 | KX212481 | KY424370 | KP289377 |
| KP144349 | KP289813 | KP144350 | KY817169 | KP005918 |
| LC421647 | KP289947 | MH539787 | KY817168 | KP005865 |
| LC364165 | KP289616 | MH539786 | MF455514 | MF962673 |
| LC364164 | KP289386 | MH539784 | MF455513 | KJ541376 |
| LC364163 | KP289871 | MH539785 | LC413181 | KJ541370 |
| LC421645 | KP289814 | KY796178 | LC413135 | KY913496 |
| LC364161 | KP289781 | KY796177 | KY497436 | KP289738 |
| LC364157 | KP289740 | KU366287 | KY817171 | KJ577348 |
| LC364153 | KP289735 | KY796183 | KY817170 | KJ577345 |
| LC364152 | KP289744 | KY796168 | MG488225 | MF962669 |
| LC364149 | KP289548 | MK167096 | MF596132 | MF962668 |
| LC364147 | KP289509 | MH111055 | MF285671 | KJ577353 |
| LC364130 | KU212264 | MH111052 | KY424376 | KJ577337 |
| LC224159 | KP289962 | MH111047 | MG488229 | KJ577333 |
| LC364132 | KJ577388 | LC420008 | KP005919 | KJ577412 |
| LC364150 | KP005893 | KY796170 | KP005874 | KJ577364 |
| LC364141 | KF836605 | MH111038 | KY424423 | KJ577366 |
| LC364143 | KY913472 | LC364185 | KT985036 | KT985028 |
| LC364142 | KJ541432 | LC364188 | KP289764 | KJ743243 |
| LC364140 | MF596129 | LC364180 | KP289827 | KJ577358 |
| LC364156 | MF285668 | LC364179 | KP289733 | KJ577357 |
| LC364159 | KY913483 | LC364178 | KP289700 | KJ577304 |
| LC364136 | KY211718 | LC364175 | KP289663 | KJ577316 |
| MH111051 | KP289789 | LC364174 | LC412938 | KJ577396 |
| KX212502 | KP289379 | LC364173 | MG385804 | KJ577322 |
| KX212415 | KU958492 | LC364172 | MF596149 | KJ577356 |
| MF596136 | KP289374 | LC364171 | MF285684 | KJ577410 |
| MF285675 | KP289872 | LC364170 | MF596148 | KJ577318 |
| KX212413 | MF596095 | LC364169 | MF285683 | KJ577310 |
| MK510082 | MF285642 | LC364176 | KY972297 | KJ577313 |
| MH111044 | MF596112 | LC364184 | KX871246 | KJ609199 |
| LC420011 | MF285654 | LC364182 | MF596111 | KJ609192 |
| LC420003 | KU708619 | LC364187 | MF285653 | KJ577376 |
| LC420002 | KU708609 | LC364186 | MF596137 | KJ577360 |
| LC420004 | KX064286 | MH111049 | MF285676 | KJ577384 |
| LC419996 | KU736932 | MH111048 | KY211709 | KJ577380 |
| KX212501 | KX064303 | MH111050 | KU708615 | KJ577397 |
| KX212422 | KU212276 | MH111032 | MF596130 | KJ609195 |
| KX212394 | KU708592 | MH111046 | MF285669 | KJ577375 |
| KX212388 | MF455512 | MH111039 | KP005909 | KJ577363 |
| KJ541375 | KJ541437 | MH111036 | KP005908 | KJ577361 |
| KX212418 | MF991294 | KY796191 | KP289727 | KJ577327 |
| KX212407 | MF962679 | KU366284 | KP289726 | KJ577406 |
| KX212381 | MF962680 | KY796190 | KF836566 | KJ577402 |
| KX212383 | KP289519 | KY796189 | KY913463 | KJ577400 |
| KX212390 | KJ541418 | KY796187 | KP005861 | KJ577321 |
| KX212376 | KJ541424 | KY796186 | KP289635 | KJ609198 |
| KX212506 | KT985037 | KY796184 | KP289782 | KJ577393 |
| KX212377 | KJ865443 | KY796182 | KU708580 | KJ577382 |
| KX212400 | MF596142 | KY796181 | KF734956 | KJ577391 |
| KX212401 | MF285679 | KY796180 | KP005930 | KJ609191 |
| KX212410 | KY856717 | KY796176 | KF836550 | KJ577359 |
| KX212409 | LC413170 | KY796172 | KF734954 | KJ577320 |
| KX212408 | LC413169 | KY796169 | KF836549 | KJ577415 |
| KX212375 | LC413168 | KU366286 | KF836548 | KJ577407 |
| KX212373 | LC413130 | KY796185 | MF596087 | KJ577386 |
| KX212396 | KY913507 | KY796175 | MF285634 | KJ577311 |
| KX212391 | KY424371 | KU366285 | MF596084 | KJ577308 |
| KX212386 | KX064285 | KY796179 | MF285632 | KJ577390 |
| KX212374 | KU736931 | KY796174 | KY424415 | KJ577385 |
| KX212419 | KX064284 | KX212340 | KP289954 | KJ577317 |
| KX212399 | KU736936 | KX212339 | KP289785 | KJ609190 |
| KX212402 | KX064283 | KJ609193 | MF596124 | KJ577354 |
| KX212387 | KP289983 | KJ577362 | MF285664 | KJ577319 |
| KX212378 | KX064299 | KJ541169 | KU708593 | KJ577339 |
| KX212420 | KU212267 | KC414754 | KU708613 | KJ577414 |
| KX212505 | KJ577373 | KC414757 | KU708614 | KJ577413 |
| KX212405 | KJ577368 | KC414751 | KU708610 | KJ577399 |
| KX212403 | KJ577371 | KC414736 | KU708601 | KJ577379 |
| KX212504 | KJ743239 | MF678318 | KU708603 | KJ577404 |
| KX212503 | KX189189 | KX212489 | KU708590 | KJ577403 |
| KX212338 | KF734955 | KX212490 | KU708600 | KC481614 |
| KX212384 | KF836561 | KX212350 | KU708597 | KC414739 |
| KX212404 | MF373606 | KC207855 | KU708598 | KY817160 |
| KX212380 | KP005882 | KY913453 | KU708596 | KY817151 |
| KX212385 | KJ541395 | KC207856 | KU708595 | KJ848324 |
| KX212379 | KC414753 | KM079533 | KU708602 | KJ848323 |
| KX212406 | KX189188 | KM079528 | KU708599 | KP289884 |
| KX212395 | KF734952 | JQ946052 | KU708591 | KP289589 |
| KX212421 | KU708573 | JQ946051 | KU708586 | KP289970 |
| KX212393 | KF734953 | JQ946054 | KU708583 | KF647885 |
| KX212392 | KC866936 | KC414729 | KT124609 | KP289762 |
| KX212398 | KJ156353 | JX495124 | KY424425 | KP289705 |
| KX212397 | MH716164 | JN582001 | KY424377 | KP289467 |
| KX212389 | MH716162 | JQ946055 | KY424413 | KP289518 |
| KX212417 | MH716163 | JQ946050 | KY424407 | KP289516 |
| KX212487 | MH018468 | MF578290 | KY424378 | MF596086 |
| KX212416 | MH018472 | MF578366 | KU708577 | MF285633 |
| KX212414 | MH018471 | MF578364 | KP289451 | MF596085 |
| KX212412 | MH018470 | MF578369 | KP289830 | KP289593 |
| KX212411 | MH018469 | MF578338 | KP005897 | KX189192 |
| LC126161 | KY817145 | MF578362 | MF596083 | KP289890 |
| LC421643 | KX064282 | MF578327 | KJ848307 | KF734958 |
| LC421644 | KU736933 | MF578331 | KJ848308 | KP289633 |
| LC421641 | MH544996 | MF578355 | KJ848306 | KP289544 |
| LC421642 | KF836598 | MF578352 | KJ848305 | KP289688 |
| LC364117 | MH544969 | MF578350 | LC412024 | KP289955 |
| AB827357 | LC012954 | MF578339 | LC013437 | KP289903 |
| LC364116 | KY211736 | MF578342 | KP289750 | KP289445 |
| LC364128 | KP005867 | MF578333 | KP289502 | KP289449 |
| LC364118 | KP005866 | MF578289 | KY424403 | KY126091 |
| LC126164 | MF962681 | MF578320 | KT985024 | KJ848318 |
| LC126163 | KJ018117 | MF578301 | KP289889 | KJ848316 |
| KY796188 | LC412041 | MF578286 | KP289772 | KJ848317 |
| KY796173 | KJ541377 | MF578285 | KP289371 | KJ848315 |
| KY796171 | KP289958 | MF578310 | KP289452 | KJ848319 |
| LC364127 | KP005846 | MF578307 | KP289788 | KY126089 |
| LC364126 | KC866972 | MF578305 | KP289482 | KY211702 |
| LC364109 | KC866956 | MF373604 | KP289476 | KY126090 |
| LC364122 | KC866948 | MF578302 | KP289594 | MF596145 |
| LC364113 | KC866946 | KX189185 | KP289538 | MF285682 |
| LC364112 | KC866933 | KM079572 | KP289532 | KY497433 |
| LC364105 | KC866927 | MF578357 | KP289530 | KY497431 |
| LC364104 | KC866926 | MF578347 | KP289511 | KF836558 |
| LC126162 | KJ541380 | MF578282 | KP289507 | KY913520 |
| LC364124 | KJ541379 | MF578376 | KJ743236 | KY913497 |
| LC364120 | MH018473 | MF578323 | KJ743238 | KJ541158 |
| LC364115 | KF836571 | MF578341 | KJ743235 | KF647891 |
| LC364125 | KF836570 | MF578380 | KP289484 | KP289996 |
| LC364123 | KF836569 | KP144339 | KP005862 | KP289922 |
| LC364121 | KF836556 | MF578373 | MF596099 | KP289881 |
| LC364119 | KY211726 | MF578325 | MF285646 | KP289603 |
| LC364114 | KF836562 | MF578358 | MF596082 | KP289779 |
| LC364111 | KP005917 | MF578372 | MF285631 | MF962671 |
| LC364106 | KF836565 | MF578346 | KJ848321 | KJ577355 |
| LC364110 | KF836552 | MF578343 | KJ848322 | KT985020 |
| LC364107 | KJ743231 | MF578344 | KJ848320 | KF647877 |
| LC364108 | KJ865461 | MF578356 | KY424397 | KP289655 |
| LC224147 | KM079581 | MF578329 | KP289491 | KM079584 |
| LC126159 | KY424400 | MF578378 | KP289828 | KJ865465 |
| LC224146 | KX189187 | MF578367 | KP289801 | KJ577309 |
| LC126160 | KF734960 | MF578353 | KP289711 | MF991291 |
| KX212507 | KC866934 | MF578359 | KP289543 | MF991290 |
| KX212382 | KP289848 | MF578349 | KP289501 | MF962664 |
| MK167105 | KP289793 | MF578336 | LC412049 | MF962663 |
| MK167082 | KP289759 | MF578326 | LC412056 | MF962665 |
| MH111035 | KP005841 | MF578345 | LC013461 | KJ577332 |
| MF596135 | KY211706 | KJ577367 | LC412054 | KY424367 |
| MF285674 | KP005894 | MH111041 | LC013459 | KY424416 |
| KX212429 | KJ541397 | MH111054 | LC412055 | KP289659 |
| KX212424 | KP289623 | MH111037 | LC412053 | KP289653 |
| KX212427 | KP289581 | MF578321 | LC013460 | KF836568 |
| KX212425 | KP289513 | MF578316 | LC013458 | KF734951 |
| KX212423 | KY424405 | MF578308 | KY211734 | KP289979 |
| MH371303 | KF836557 | MF578283 | LC013449 | KP289971 |
| KX212508 | KF836554 | MF578381 | KY211733 | KP289925 |
| KX212428 | KJ865462 | MF578330 | KU212270 | KP289964 |
| KX212426 | MH544988 | MF578332 | KX064305 | KP289915 |
| KX212488 | MH544973 | LC412023 | KP289368 | KP289913 |
| KX212483 | KP005856 | KY211729 | KM079580 | KP289933 |
| KX212485 | KP005844 | MF578340 | MH018498 | KP289941 |
| KX212484 | KP005843 | MF578288 | MH018500 | KP289722 |
| MF596072 | KC866939 | KX430800 | MH018497 | KP289668 |
| LC420007 | KC866940 | KX430799 | MH018499 | KP289666 |
| LC364167 | KP005855 | MF578295 | LC481414 | KP289638 |
| LC364166 | KP005854 | MF578312 | LC481413 | KP289497 |
| LC364191 | KP005851 | MF578293 | LC481404 | KP289654 |
| LC364192 | KP005849 | KX212372 | LC481412 | KP289981 |
| LC364190 | KP005848 | MF578348 | LC481410 | KP289878 |
| LC364177 | MH544977 | MF578303 | MG385762 | KP289834 |
| LC421654 | KT985018 | MF578328 | LC481432 | KP289802 |
| LC364168 | KC866945 | MF578370 | LC481409 | KP289767 |
| LC364189 | KC866969 | MF578324 | LC481397 | KP289692 |
| LC364193 | KC866942 | MF578360 | LC413131 | KP289660 |
| LC364162 | KC866937 | MF578363 | KY817178 | KP289823 |
| LC421653 | KY211693 | MF578311 | KY972298 | KP289944 |
| LC419998 | KT985030 | MF578287 | KY972296 | KP289911 |
| LC420012 | MH049746 | MF578294 | KX871247 | KP289770 |
| LC419997 | KP005875 | KX430796 | KY211735 | KP289776 |
| LC421655 | KJ784508 | KX430795 | MF596138 | KP289701 |
| LC420009 | KJ577395 | MF578377 | MF596127 | KP289684 |
| LC224160 | KJ577394 | MF578284 | MF596131 | KP289551 |
| LC364183 | KT985038 | MF578354 | MF285670 | KP289939 |
| LC364181 | KP289990 | MF578337 | MH716165 | KP289658 |
| MH111033 | KJ541427 | MF578375 | KY424375 | KF647878 |
| LC420010 | KJ541426 | MF578351 | MH716168 | MF596077 |
| LC224161 | MH018466 | MF578335 | MH018496 | MF285627 |
| MF596128 | MH018467 | KX430802 | MG488227 | KY424388 |
| MF285667 | KR815992 | KX430801 | MG457757 | KJ743241 |
| LC224154 | KC866980 | MF578334 | KY424363 | KJ577370 |
| LC224153 | KY913455 | MF578291 | KY424362 | KC481616 |
| LC364151 | MH018465 | MF578371 | KY424361 | KC414727 |
| LC364160 | MH018464 | MF578317 | KY424360 | KJ577342 |
| LC364148 | KU708575 | MF578304 | KY424372 | KJ577341 |
| LC364137 | KJ577334 | MF578309 | KY211714 | KC414749 |
| LC364138 | MF962667 | MF578299 | KY211704 | KC414740 |
| LC364135 | KJ541429 | MF578313 | KY211703 | KC207843 |
| LC364134 | KJ541159 | MF578368 | KY126092 | KP289526 |
| LC364158 | KT124606 | MF578300 | KY817157 | KP289753 |
| LC364155 | MF596074 | MF578292 | MH716166 | KP289584 |
| LC364154 | KP289681 | MF578315 | KY497430 | KJ743225 |
| LC364146 | KP289677 | MF578314 | KY913499 | KF647886 |
| LC364145 | KP289546 | MF578297 | KY913498 | KC414750 |
| LC421646 | KP289558 | MF578298 | KY972308 | KC207833 |
| KF836581 | KC414731 | MF578365 | KY913506 | KC207834 |
| MF596134 | KC207852 | MF578296 | KY913482 | KC207837 |
| MF285673 | KJ577306 | MF578319 | MH018479 | KC207840 |
| KX595286 | KY424409 | MF578322 | KY856713 | KC207842 |
| LC224158 | KC866968 | KX430798 | KY817164 | KC207844 |
| LC224157 | KC207838 | KX430797 | LC412960 | KC207850 |
| LC224152 | KY913476 | MF578361 | LC412959 | KC207851 |
| LC224151 | KJ848303 | MF578374 | LC412958 | KC414726 |
| LC224149 | KJ848302 | MF578306 | LC412951 | KC414728 |
| LC224148 | KF836563 | MH111040 | LC412949 | KC414730 |
| LC224156 | KT985029 | KJ743240 | LC412956 | KC414738 |
| LC224150 | KP289642 | KP289676 | LC412950 | KC414746 |
| KY796192 | KP289474 | KY424422 | KY817163 | KC414748 |
| KY796166 | KY211691 | KF647882 | KY817162 | KC481617 |
| KY796164 | MH544987 | KY424417 | KY817155 | KJ743223 |
| KY796163 | KJ577408 | KP289463 | KY817161 | KJ743216 |
| KY796161 | KJ577335 | KP005850 | KY817152 | KJ541441 |
| KY796160 | KJ577336 | KP005900 | KX064296 | KJ541157 |
| KY796157 | KY913445 | KP005872 | KU736946 | KJ577398 |
| KY796165 | KY913444 | KP289997 | KY913488 | KJ577383 |
| KY796162 | KT985031 | KP005892 | MH018478 | KJ541393 |
| KY796156 | KC414734 | MF962670 | KX364096 | KJ577411 |
| KY796167 | KC866957 | KP005911 | KX364095 | KP289683 |
| KY796159 | KY913446 | KP005910 | KX364094 | KP289629 |
| KY796158 | KJ577302 | KP005871 | KY913508 | KJ577350 |
| KX212434 | KJ577374 | KP005863 | KY913523 | KJ865463 |
| KX212433 | KP289824 | KP005907 | KY913510 | KY424418 |
| KX212432 | KJ577416 | KP005906 | KY913509 | KJ577381 |
| KX212431 | KY913450 | KP005903 | KY817185 | KJ577352 |
| KX212430 | KY913442 | KP005889 | LC413126 | KP289924 |
| KP144343 | KP005847 | KP005860 | LC361279 | KP289992 |
| KX212435 | KC207854 | KP005870 | KY817177 | KP289975 |
| KX212509 | KC867007 | KP005912 | KY817166 | KP289927 |
| KJ541438 | KC866960 | KP005873 | KY817186 | KP289874 |
| KC207845 | KC414741 | KY424389 | MG385812 | KP289496 |
| KJ541439 | KX189191 | KP289514 | MG385811 | KJ743220 |
| KJ865442 | KF734957 | MF962666 | KY913503 | KJ577338 |
| KC414745 | KJ577307 | LC412013 | LC413127 | KJ848309 |
| JX473336 | KJ577351 | LC013416 | KY817154 | KJ743230 |
| KF682363 | KM079549 | LC412963 | KY424369 | KP289731 |
| KJ018118 | KC866998 | LC412962 | KY497435 | KP289867 |
| MF962674 | KC866970 | KX871245 | KY913522 | KP289850 |
| MF596081 | MH544999 | KX871244 | KY913501 | KP289715 |
| MF285630 | MH544998 | KX871242 | KY913500 | KP289628 |
| KF836593 | MH544993 | KU736940 | KY211712 | KP289689 |
| KC866963 | MH544980 | MG488223 | KY424387 | KP289486 |
| KC867010 | KY913451 | MG488221 | MG385748 | KJ743214 |
| KC866996 | KY913458 | LC412953 | LC413182 | KP289885 |
| KC866932 | KY913457 | LC412946 | MH018494 | KP289783 |
| KC866931 | KY913447 | KY913528 | MH018493 | KP289769 |
| KC866930 | MH544983 | KY913515 | KY972307 | KP289857 |
| KC866993 | MH544997 | KY913505 | MH018491 | KP289475 |
| KC866973 | MH544974 | KY211717 | LC361280 | KP289747 |
| KC866935 | MH544995 | KU736941 | KY913492 | KP289598 |
| KF836587 | MH544976 | KY211700 | MH018492 | KP289572 |
| KC866944 | MH544986 | KU212266 | KX595285 | KP289527 |
| KC866929 | MH544978 | MH049747 | MH018495 | KP289712 |
| KC866941 | MH544989 | MG385790 | MH049744 | KP289461 |
| KC866977 | KJ743229 | MG385763 | LC481393 | KP289743 |
| KY913469 | MH544994 | MG385758 | LC413179 | KP289573 |
| KY913462 | KY913456 | MG385771 | LC413177 | KP289555 |
| KY424394 | MH544972 | MG385766 | LC413176 | MF596097 |
| KJ541386 | KP289576 | MG385760 | LC413175 | MF285644 |
| KJ541385 | KY424421 | MG385755 | LC413173 | KY913470 |
| KJ541384 | KC414733 | MG385789 | LC413138 | MH018480 |
| KJ541374 | KC207846 | MG385779 | LC413178 | KJ865455 |
| KJ541165 | KC207847 | MG385783 | LC413174 | MH018481 |
| KJ541156 | KP005845 | MG385785 | LC413172 | KP289998 |
| KJ541155 | KC207841 | MF596133 | LC412966 | KU708579 |
| KJ541154 | KC207836 | MF285672 | LC412964 | KT124608 |
| KF647892 | KJ784509 | KY972299 | LC412957 | KP289918 |
| KP289390 | MH544975 | LC413167 | LC412948 | KP289904 |
| KF836596 | KY913452 | KJ865460 | LC412954 | KP289821 |
| KC866952 | KY913459 | MF596101 | LC413164 | KP289862 |
| KY211725 | MH545001 | MF285648 | LC413153 | KP289897 |
| KJ577303 | MH545000 | KX871239 | LC413163 | KP289768 |
| KF647876 | MH544990 | MH018501 | LC413162 | KP289907 |
| KC207849 | KY913443 | MH049748 | LC413159 | KP289887 |
| KC207848 | KP289656 | MH018503 | LC413150 | KP289869 |
| KF836588 | KP289535 | MH018502 | MH018487 | KP289893 |
| KC866949 | KJ743222 | KY211715 | KJ577331 | KP289877 |
| KF836583 | KJ743221 | KX752785 | KP005928 | KP289752 |
| KC867000 | KC414742 | KY211737 | KP005920 | KP289568 |
| KC866997 | MH544991 | KY211713 | KX064291 | KP289940 |
| KC866987 | KJ743211 | KY211689 | KP005902 | KP289916 |
| KC866978 | KP289498 | KY211708 | KF836604 | KP289856 |
| KC866975 | KP289626 | MF578379 | KP289741 | KY211692 |
| KC866943 | KP289393 | KP289730 | KP289695 | KP289621 |
| KC866923 | KP289366 | KY913466 | KM079583 | KP289549 |
| KC414752 | KP289365 | KP289780 | KF836560 | KP289483 |
| KC866991 | KU708616 | KP289579 | KM079582 | KP289455 |
| KC866984 | KJ541436 | KP289945 | KF836559 | KP289946 |
| KC867003 | KC414735 | KP289554 | KY817156 | KP289932 |
| KC866982 | KJ743228 | KP289545 | MF596144 | KP289679 |
| KC866922 | KP005901 | KP289539 | MF285681 | KP289914 |
| KP005857 | MH018463 | KP289536 | MF596147 | KP289832 |
| KF836551 | MH018462 | KP289456 | MF596146 | KP289488 |
| KC866976 | KJ018113 | KP289447 | MF596141 | KP289485 |
| KC866938 | KJ865445 | KP289667 | MF285678 | KT779413 |
| KC866995 | KF836594 | KP289508 | KY424373 | KP289952 |
| KC866986 | KY913448 | KP289563 | KY211697 | KP289994 |
| KC866990 | KJ865452 | KP289376 | KJ743224 | KP289988 |
| KC866988 | KF836600 | KP289625 | KX189193 | KP289966 |
| KC866999 | KF836586 | KP289591 | KF836553 | KP289934 |
| KC866994 | MH544967 | KP289755 | MF596091 | KP289905 |
| KC866992 | MG018992 | KP289728 | MF285638 | KP289929 |
| KC866951 | KJ743210 | KP289590 | KY972306 | KP289886 |
| KY424359 | KC866967 | KP289634 | KP005929 | KP289672 |
| KC867001 | KM079579 | KP289820 | KP005927 | KP289829 |
| KC866955 | KC866961 | KP289720 | KP005925 | KP289980 |
| KC866928 | MH018461 | KP289716 | KM079585 | KP289852 |
| KC866965 | KM079554 | KU708576 | KP005899 | KP289714 |
| KC867004 | KJ865436 | KF647879 | KP005883 | KP289861 |
| KP005933 | KJ865435 | KJ865453 | LC412057 | KP289647 |
| KP005924 | KJ865432 | KP289853 | KJ018116 | KP289450 |
| KP005923 | KJ865431 | KP289773 | LC364144 | KP289524 |
| KP005886 | KM079562 | KP289664 | KJ577365 | KP289531 |
| KJ865457 | KC866954 | KP289570 | MK167100 | KP289540 |
| KF836592 | KY424410 | KP289459 | MK167101 | KP289600 |
| KF836589 | KX189179 | KP289458 | MK167115 | KP289601 |
| KC481615 | KM079561 | KX189194 | MK167090 | KP289605 |
| KF836591 | KF836582 | KF734959 | MK167046 | KP289373 |
| KP005877 | KC867009 | KF647890 | MK167109 | KP289521 |
| KJ865464 | KC866979 | KP289791 | KJ541435 | KP289505 |
| KJ577346 | KC866953 | KP289775 | MH111042 | KP289466 |
| KJ577344 | JX473397 | KP289811 | MH111043 | KP289499 |
| KC866989 | KX189178 | KP289641 | MH111034 | KJ577329 |
| KC866924 | KM079553 | KP289528 | LC412942 | KJ577409 |
| KP005842 | KM079555 | KM079587 | KJ743218 | KJ577349 |
| KC866964 | KF836584 | KF836606 | KJ743217 | KJ577325 |
| KC866962 | KY913441 | KP005914 | KU958493 | KJ577328 |
| KC866925 | KM079551 | KP005913 | KP289381 | KJ577326 |
| KC414756 | KM079529 | KF682362 | KP289669 | KP005931 |
| KC866983 | KM079527 | MF596114 | KP289478 | KX212492 |
| KC867006 | KM079546 | MF596113 | KP289883 | KX212370 |
| KP289388 | KM079545 | MF285656 | KP289873 | KX212369 |
| KF836564 | KM079544 | MF285655 | KP289808 | KX212368 |
| KF836602 | KJ865437 | KY211721 | KP289632 | KX212361 |
| KP005932 | JX473353 | KX064294 | KP289699 | KX212364 |
| KF836573 | JX473339 | KU736944 | KP289662 | KX212359 |
| KY913431 | KM079543 | KJ541388 | KP289661 | KX212358 |
| JX473370 | KM079542 | MF596096 | KP289523 | KX212360 |
| KJ865454 | KM079520 | MF285643 | KP289522 | KX212356 |
| KF836599 | KM079530 | KY424382 | KP289839 | KX212366 |
| LC421639 | KJ577296 | KP289704 | KP289645 | KX212367 |
| AB678778 | JX473394 | KP289646 | KP289665 | KX212357 |
| AB649288 | KC866950 | KY424404 | KP289583 | KX212362 |
| AB649287 | JX473398 | KY424365 | KJ848314 | KX212363 |
| AB649289 | JX473405 | KY424392 | KP289674 | KX212355 |
| AB649286 | KT985008 | KJ865456 | KJ848313 | MH111045 |
| AB649291 | KJ018115 | KY424414 | KT985027 | LC364139 |
| LC421640 | KC414755 | KP005868 | KT779411 | LC364131 |
| LC224139 | KY424396 | MF596115 | KT985013 | LC364133 |
| LC224136 | MH544985 | MF285657 | KJ541391 | LC364129 |
| LC224138 | KX575649 | KP289978 | KJ541369 | LC224155 |
| LC126153 | KM079576 | KP289826 | KP289931 | KX212486 |
| LC126158 | KJ865439 | KP289921 | KP289462 | KX212371 |
| LC224144 | KP005869 | KP289795 | KP289378 | KX212352 |
| LC224143 | KP005859 | KP289618 | KP289686 | KX212365 |
| JX495149 | KJ865450 | KP289725 | KP289552 | KX212354 |
| JX495138 | KF836574 | KP289610 | KP289525 | KX212353 |
| LC224145 | KJ577324 | KP289757 | KP289541 | KX212494 |
| LC224141 | MF962672 | KP289643 | KP289578 | KX212493 |
| LC224140 | KC414747 | KP289542 | KP289577 | KP005926 |
| LC224137 | KJ743237 | KP289737 | KP289574 | KP005879 |
| LC224135 | KF836597 | LC012947 | KP289571 | KM079586 |
| LC126156 | JX495144 | LC012946 | KP289567 | MF596116 |
| LC224142 | KJ541166 | LC012945 | KP289566 | MF285658 |
| LC126157 | JX495123 | KY913468 | KP289565 | KX064307 |
| LC126155 | KY913454 | KX064293 | KP289564 | MF596088 |
| LC126154 | KC414737 | KX064292 | KP289562 | MF285635 |
| JX495128 | KC414744 | KU212261 | KP289627 | KY211698 |
| KY211738 | KC207853 | MF596105 | KP289624 | KJ848311 |
| KX212437 | KJ865451 | MF285651 | KP289471 | KJ848310 |
| KX212436 | KJ865448 | KU708608 | KP289953 | KJ848312 |
| KM114057 | KC866974 | KU708607 | KP289950 | KX064308 |
| HE572937 | KF836595 | KU708604 | KP289984 | KY972295 |
| HE572914 | KJ865438 | KU708605 | KP289899 | KX871248 |
| HE572913 | KF836575 | KU708618 | KP289760 | KU736945 |
| HE572906 | KJ865433 | KU708594 | KP289736 | KY817180 |
| HE572903 | JX495148 | KU708588 | KP289723 | KY817184 |
| HE572925 | MH018474 | KU708587 | MG488230 | KY817176 |
| HE572902 | KY913438 | KU708585 | MG488222 | KY817181 |
| HE572905 | JX473409 | KU708584 | KY424368 | KX364093 |
| HE572907 | KF836585 | KU708582 | KP289739 | KP005895 |
| HE572904 | KF836576 | KT985035 | KP289611 | KP005887 |
| KP144346 | KJ865446 | KT985022 | KP289561 | KP005885 |
| KP144345 | KJ577378 | KT985017 | KP289557 | KP005880 |
| KP144344 | KT985021 | KU708617 | KJ743233 | KP005878 |
| HE572909 | KT985034 | KP289963 | KJ541434 | KP005890 |
| KX212500 | KT985009 | KP289937 | MH716171 | KP005858 |
| KX212346 | KJ865449 | KP289804 | KY913519 | KP005884 |
| KX212499 | KC866966 | KY913467 | KY817150 | KX212491 |
| KX212345 | KC866958 | KP289806 | KY817144 | KP289923 |
| LC126150 | KC866959 | KP289687 | MF596118 | MH018485 |
| AB779618 | KJ156352 | KP289982 | MF285660 | KY913471 |
| AB779615 | KJ156351 | KP289930 | LC412947 | KX064295 |
| KX212498 | KJ784510 | KP289809 | KY817149 | KP289690 |
| KX212344 | KJ156349 | KP289707 | KP289928 | KP289517 |
| KX212496 | MH049745 | KP289721 | KP289985 | KP289469 |
| KX212495 | KC867005 | KP289602 | KP289810 | MF596080 |
| KX212342 | KC866971 | KP289614 | KP289756 | MF285629 |
| KX212341 | JX495125 | KP289774 | KP289745 | KU708578 |
| KX212497 | MH018477 | KP289671 | KP289675 | KP005905 |
| KX212343 | KJ609187 | KP289631 | KP289777 | KP005904 |
| JX154930 | KJ577286 | KP289500 | KP289718 | KP005891 |
| KM079523 | KJ743208 | KP289693 | KP289882 | KP005888 |
| KC866907 | KM079539 | KP289595 | KP289960 | KP005864 |
| KJ577295 | KM079538 | KP289698 | KP289892 | KP289959 |
| KJ577278 | KM079537 | KP289680 | KP289854 | KP289748 |
| JN655885 | KM079536 | KP289547 | KP289797 | KP289800 |
| KJ577294 | KM079521 | KP289697 | KP289784 | KP289766 |
| KJ577277 | KJ541168 | KP289630 | KP289670 | KP289369 |
| KJ577291 | KM079517 | KP289608 | KP289637 | KJ541433 |
| JN655887 | KX189181 | KP289537 | KP289533 | KP289858 |
| KJ577298 | KM079564 | KJ541368 | KP289529 | KP289864 |
| KJ577279 | KM079552 | KP289948 | KJ577330 | KP289596 |
| JN655884 | JX154938 | KP289636 | MH018486 | KJ743242 |
| KF836766 | KM079565 | KP289464 | MF596123 | KP289993 |
| KF836765 | JX473404 | KP289575 | MF285663 | KT985025 |
| JX154922 | KM079556 | KP289493 | MF596120 | KP289969 |
| KY913465 | KJ865441 | KP289370 | MF285662 | KU212278 |
| KX189184 | JX495147 | KP289754 | KY817158 | KY211728 |
| KM079570 | JX495143 | KP289504 | MF596108 | KF836767 |
| KX189182 | JX495140 | KP289613 | KY972302 | KJ577347 |
| KM079560 | JX495146 | KP289560 | KU212277 | KP289866 |
| KM079566 | KM079574 | KP289495 | MF596104 | KU958490 |
| KJ865440 | KC867002 | KP289480 | MF285650 | KP289367 |
| JX473351 | JX473333 | KP289372 | KY913487 | KP289612 |
| KX189183 | KC866981 | KP289481 | KY913485 | KP289609 |
| KM079569 | KF836590 | KP289448 | KY913516 | KP289550 |
| MF596071 | JX495131 | KP289559 | KU736938 | KP289710 |
| MH018476 | JX154933 | MF596094 | LC013469 | KP289765 |
| KC866985 | JX154932 | MF285641 | KY211740 | KP289870 |
| KC866947 | KY913428 | KP289763 | KY211716 | KP289909 |
| KJ577314 | KJ865430 | KP289639 | MF596125 | KP289799 |
| KC207835 | KY913427 | KP289972 | MF285665 | KP289794 |
| KJ609189 | KJ865428 | KP289831 | KU708589 | KP289805 |
| KJ577343 | JX154935 | KP289622 | KU708612 | KJ577372 |
| KC414732 | KM079548 | KP289553 | MF596119 | KJ577369 |
| KM079568 | KM079518 | KP289490 | MF285661 | LC012953 |
| KM079567 | KJ865426 | KP289487 | MF596117 | KP289644 |
| JX495142 | KJ577275 | KY913481 | KY211722 | KP289961 |
| JX473371 | KJ577274 | KP289995 | MF285659 | KP289896 |
| MH018475 | KJ577276 | KP289949 | KY913477 | KP289822 |
| KJ848304 | KM079515 | KP289894 | KY913475 | KP289746 |
| KJ743226 | KY913435 | KP289835 | KJ577401 | KP289841 |
| KC207839 | KJ865423 | KP289790 | KX064290 | KP289729 |
| KJ743227 | JX154956 | KP289696 | KP289999 | KP289473 |
| KY913437 | JX154953 | KP289901 | KP289965 | KJ609197 |
| KJ541167 | KM079525 | KP289815 | KP289957 | KJ577323 |
| JX495122 | KM079524 | LC481394 | KP289968 | KJ609196 |
| KX189186 | KM079514 | LC481423 | KP289859 | KJ609194 |
| KM079575 | KJ865425 | LC481421 | KP289713 | KJ577377 |
| JX495136 | JX495139 | MF596150 | KP005876 | KJ743234 |
| JX495135 | KC866910 | LC481431 | KY913524 | KY424399 |
| JX495121 | KC866920 | LC481430 | KY913502 | KP289976 |
| JX154931 | KM079505 | LC481405 | KY913494 | KP289506 |
| KM079573 | KX212349 | KY231153 | KY211741 | KP289751 |
| KM079535 | KX212348 | KY231155 | KX871238 | KF836572 |
| KM079534 | KP144342 | KY231154 | KU736937 | KY913460 |
| KP289586 | KY424424 | MH111053 | KY211720 | KY424402 |
| KP289494 | KP005853 | MG385808 | KJ541372 | KJ577305 |
| KY913473 | KP005852 | MG385792 | KX064289 | KP289384 |
| KU958494 | KJ865459 | MG385750 | LC412058 | KP289453 |
| KP289383 | KJ865458 | KY817179 | LC013471 | KP289454 |
| KP289868 | KX189180 | MG385831 | KY211739 | KP289465 |
| KP289863 | KM079563 | LC413124 | LC013470 | KP289556 |
| KP289489 | KM079571 | MG385768 | KY913489 | KP289585 |
| KP289382 | KM079550 | LC481403 | KY424379 | KP289592 |
| KP289678 | KY913432 | MG385794 | MF596110 | KP289607 |
| KP289619 | KM079547 | MH018504 | KY913493 | KP289732 |
| KP289512 | MH018460 | MG385809 | KY424364 | KP289749 |
| MH544992 | KJ865434 | MG385769 | KY913490 | KP289778 |
| MH544979 | KF836580 | MG385780 | KY424383 | KP289787 |
| MH544968 | KF836577 | MG385777 | KY424385 | KP289803 |
| KY913449 | KF836578 | MG385761 | KY424380 | KP289846 |
| MH544971 | KM079541 | MG385770 | LC412014 | KP289912 |
| KJ541387 | JX154943 | MG385756 | KP289758 | KP289920 |
| KT985033 | KP289986 | KP289977 | KP289973 |  |

| **CV-A10 Sequences** | | | | |
| --- | --- | --- | --- | --- |
| LC412004 | KU885542 | LC412005 | KX768159 | FR796493 |
| LC412009 | LC412003 | MG838846 | KX768160 | FR796479 |
| LC412010 | LC013452 | KP005987 | KY913591 | LC430225 |
| LC412008 | LC013450 | MG838797 | KY913588 | LC430226 |
| LC013467 | LC013468 | KM048116 | KX768198 | LC411993 |
| LC013466 | LC412011 | KM048120 | KY913595 | KX430810 |
| LC013465 | MG838881 | KM048117 | MH111065 | KX430803 |
| LC013412 | LC411997 | KM048119 | MH111066 | KX430804 |
| LC411987 | LC411998 | KM048113 | MH111064 | KX430805 |
| LC013418 | LC411999 | KM048118 | MH111063 | KX430806 |
| LC411996 | KF999774 | LC412007 | MH111060 | KX430807 |
| MG838796 | JX154970 | MG838882 | MH111059 | KX430808 |
| MG838849 | KM048103 | MG838785 | MH111058 | MF678312 |
| KY913580 | KF999730 | KM048121 | MH111062 | MF422532 |
| KU885571 | MG838813 | KU578128 | KU578135 | KC879534 |
| KP009581 | KP289402 | KU578129 | MG838864 | GQ214176 |
| KF999776 | KP289406 | KU578133 | MF596048 | MG838802 |
| LC411990 | KP289395 | MG838831 | KU578131 | GQ214175 |
| KU578130 | KP289397 | MG838863 | KY272008 | GQ214177 |
| LC167417 | KP289400 | MG838853 | MG838852 | KC867028 |
| KM048112 | KP005985 | MG838847 | KY913600 | MF422531 |
| KP289401 | MG838787 | MG838789 | MG838800 | GQ214174 |
| MH716177 | KJ641623 | KY913578 | KY913581 | GQ214172 |
| KX595290 | MG838880 | MG838862 | MG838860 | KC867026 |
| MH716178 | KP861810 | KF999784 | MG838866 | KF999747 |
| MG838791 | MG838869 | KY913576 | KU578132 | JN255588 |
| LC013413 | KU578136 | MG838781 | KX768192 | JX307651 |
| LC411988 | KY272010 | MF596064 | MG838868 | KC879491 |
| LC412002 | MG838821 | KX768190 | KY913589 | KC879488 |
| LC013430 | KY913566 | KX768191 | KP289407 | MH118054 |
| LC412000 | KC867030 | KX768156 | KX768203 | MH118044 |
| KJ156357 | KC867025 | KX768185 | KX768161 | MH144603 |
| MG838819 | KC867024 | KX768189 | MG838857 | MH118048 |
| KP289405 | KC867029 | KX768186 | KU885565 | MH118058 |
| MF596042 | KC867022 | KX768188 | MF596044 | MH118065 |
| JX473458 | KF999748 | MG838809 | MG838850 | MH118076 |
| JX473459 | KF999749 | KU885570 | MG838838 | MH118071 |
| KF246668 | KF999750 | KU885572 | KY913601 | MH118057 |
| KF246670 | KF999746 | KU885557 | LC013474 | MH118059 |
| KF246667 | KU885552 | KJ784512 | LC412012 | MH118061 |
| MF137166 | MG838812 | KY913583 | MF596057 | MH144595 |
| KF999738 | KY913532 | KJ156359 | KX768199 | MH118085 |
| KF999735 | KY913535 | KU885553 | KX768167 | MH118089 |
| KF999734 | KF999731 | MG838783 | LC412978 | MH118066 |
| KY913562 | KP009580 | KF246660 | MG838803 | MH144591 |
| JX473453 | JX473449 | KF246661 | MG838830 | MH118056 |
| KC867040 | KF999775 | KF246663 | MG838836 | MH118083 |
| KX768180 | KF999762 | KF246659 | MG838818 | MH118049 |
| KX768182 | KC867034 | KF246662 | MF596054 | MH144599 |
| KY012321 | KC867033 | MG838807 | MF596056 | KC879497 |
| KU885549 | JX154976 | MF596043 | KY913538 | KC879487 |
| KP005986 | JX154978 | KX768200 | KC867027 | KF413053 |
| KY913547 | MF596029 | KX768158 | KX768171 | MH118072 |
| KC867039 | KM048107 | KX768187 | KX768173 | MH144592 |
| KY913568 | MF596031 | JX473460 | KX768170 | MH118069 |
| MG838788 | MF596033 | JX473455 | KX768172 | MH118055 |
| KY913571 | MF596030 | KF999764 | JQ320273 | MH144596 |
| KY913575 | KY913573 | KF999765 | JQ320248 | MH118053 |
| JX154982 | KY913579 | MF688814 | MF596039 | MH118070 |
| JX154981 | MG838872 | KX768181 | KC867038 | MH118077 |
| JX154979 | KU885562 | KJ784511 | KF999780 | MH118074 |
| KY913559 | KP289399 | MG838835 | KF999782 | MH118081 |
| KJ156354 | KP289403 | MG838832 | MF137169 | MH118080 |
| KY913555 | MG838865 | MG838799 | MF137168 | MH118088 |
| KY913556 | KU885569 | MG838843 | GU947774 | KF412913 |
| KY913553 | KU885564 | MG838817 | GU947775 | MH118060 |
| KY913534 | KU885568 | MF596051 | KF999783 | MH118084 |
| KF999737 | KP289404 | MF596046 | KU885543 | MH118046 |
| KF246664 | KF999781 | MF596049 | KY913537 | MH118050 |
| KF246665 | MG838871 | KX595288 | KF999768 | MH118052 |
| KF999778 | KF999786 | MG838790 | KF999785 | MH118062 |
| KF246669 | GU947787 | MH716176 | KX768174 | MH118063 |
| KF246666 | JX154972 | KX768193 | KX768169 | MH118075 |
| KY913569 | KY913554 | KX768168 | KC867021 | MH118079 |
| KY913550 | KM048115 | KU885566 | KX768177 | MH118087 |
| KY913561 | JX154980 | KU885567 | KC867020 | MH118090 |
| KY913542 | JX154973 | MG838828 | KY913540 | MH118067 |
| KF999771 | KY913546 | KU578134 | KM048104 | MH118073 |
| KY913536 | KY913549 | KU578127 | MF596035 | MH118078 |
| KF999767 | KY913539 | KY272009 | MF596036 | MH118082 |
| KF999770 | KF999757 | KY913593 | KM048105 | MH118086 |
| KF999766 | KF999772 | MG838784 | KC867042 | MH118068 |
| KY913548 | KC867031 | MF596050 | KY913541 | MH118047 |
| KY913551 | JX154977 | MG838794 | KJ784513 | MH118064 |
| KC867035 | KY913545 | MG838826 | KY913564 | MH118045 |
| MG838878 | KF999769 | KY913590 | JX154975 | MH118051 |
| MG838879 | KY913565 | KY913596 | KF999754 | MH118043 |
| MG838840 | KF999739 | MF596060 | KF999758 | MH118023 |
| KU885561 | KJ156358 | MG838855 | JQ320252 | MH118042 |
| KU885560 | KX768175 | MG838810 | KX768197 | MH118039 |
| JX154971 | KX768176 | MG838844 | GU947782 | MH144590 |
| KF999756 | KX768179 | MG838851 | GU947786 | MH118036 |
| KF999743 | KX768196 | MF596055 | GU947777 | MH144593 |
| KF999740 | KX768157 | MF596045 | GU947781 | MH118034 |
| KF999741 | MF596041 | KX768195 | GU947783 | MH118037 |
| KF999742 | KY913557 | KX768165 | GU947779 | MH118038 |
| KF999753 | MG838876 | KX768209 | GU947776 | MH118040 |
| JX473461 | MF596061 | KX768206 | GU947780 | MH118033 |
| JX473462 | MG838859 | KX768163 | GU947778 | MH118035 |
| KY913558 | KP289408 | LC430231 | GU947785 | MH144594 |
| KY913544 | KP289394 | LC430232 | GU947784 | MH144597 |
| KJ156355 | KP289409 | LC430230 | KC867023 | AY421767 |
| KT588920 | MG838884 | LC430228 | KF999751 | AF081300 |
| KP009575 | MG838814 | LC430234 | GQ214173 | KC879507 |
| KP009577 | MG838833 | LC430235 | KC867037 | MH118041 |
| KP009574 | KP289396 | LC430236 | JX473450 | KR185980 |
| KP009576 | KP289398 | LC430227 | KX768178 | KC879528 |
| KP009579 | KY913587 | LC430233 | MG838870 | KC879535 |
| KF999763 | MG838825 | LC481439 | KF999752 | HE572947 |
| KU885544 | KY913582 | LC481435 | KF999755 | HE572951 |
| JX473452 | KU885559 | LC481437 | MF596027 | HE572967 |
| MG838824 | LC411994 | LC481438 | KY913530 | HE572956 |
| MG838820 | LC411992 | LC481436 | KC867032 | HE572973 |
| KP289410 | LC411991 | MG838816 | KY082004 | HE572949 |
| MG838845 | KF999759 | MG838874 | KF246675 | HE572965 |
| MF596040 | KF999760 | KY913597 | LC167415 | HE572970 |
| KC867041 | MG838805 | LC412979 | KC867018 | HE572971 |
| KF999761 | MG838841 | MG838854 | KC867019 | HE572957 |
| KX595289 | KU885556 | KY913602 | KF999779 | HE572988 |
| KC867043 | KU885555 | LC412983 | MF137167 | HE572976 |
| KU885550 | KU578126 | LC412985 | KF246672 | HE572978 |
| KF999773 | KY272007 | MG838848 | KF246677 | HE572984 |
| KP009578 | KF999745 | LC412987 | KF246678 | HE572964 |
| KY913560 | LC361284 | KY913598 | KF246674 | HE572961 |
| KU885546 | LC361285 | KY913599 | KF246673 | HE572948 |
| KU885545 | MG838861 | MF596062 | KY082003 | HE572969 |
| KU885547 | MG838837 | KX768205 | MF596034 | KC879537 |
| KM048108 | MF596059 | MG838873 | KY913543 | KC879518 |
| KF999736 | MF596058 | KY913603 | KF246671 | HE572944 |
| KM048111 | MG838792 | MF596063 | KY913533 | FR796483 |
| KF999733 | MF596052 | MG838839 | HQ728262 | HE572987 |
| KU885551 | LC481434 | MG838808 | MF596037 | HE572953 |
| KY913531 | LC412986 | LC120872 | MF596038 | HE572940 |
| JX473456 | MG838795 | LC120873 | MG838801 | KC879515 |
| MF596032 | LC412984 | LC167416 | MF596028 | HE572955 |
| KY913552 | MG838811 | LC120874 | KF246676 | HE572963 |
| JX473446 | MG838782 | MF596047 | KF999744 | HE572982 |
| JX473447 | MG838827 | MG838883 | KF150147 | HE572985 |
| JX473451 | MG838786 | LC412001 | KF150148 | HE572989 |
| KY913567 | MG838877 | KY913586 | KF150149 | HE572991 |
| MF137165 | MG838856 | MG838834 | MG838804 | HE572977 |
| JX473454 | KX768208 | KX768204 | KM048114 | HE572992 |
| KX768183 | KX768166 | KX768162 | KM048109 | HE572979 |
| KX768184 | MG838842 | KU885563 | FR796489 | HE572983 |
| KY913570 | LC013463 | MH111061 | FR796490 | HE572975 |
| KC867044 | LC412006 | MG838829 | FR796491 | HE572941 |
| KC867036 | LC013415 | MG838875 | HE572990 | HE572981 |
| KY913585 | LC411989 | KY913592 | FR796482 | HE572946 |
| KY913577 | KM048110 | KY913594 | FR796478 | HE572958 |
| MG838823 | KY913563 | MF596053 | FR796480 | HE572945 |
| KY913572 | MG838822 | KY271944 | FR796476 | HE572968 |
| KX595287 | KY913574 | MG838867 | FR796477 | HE573016 |
| KU885558 | KF999732 | KX768194 | FR796481 | HE572974 |
| KU885554 | MG838858 | MG838798 | FR796484 | HE572986 |
| KF999777 | KM048106 | KY913584 | FR796485 | HE572952 |
| KU885548 | MG838815 | KX768201 | FR796486 | HE572943 |
| KU885541 | MG838793 | KX768202 | FR796487 | HE572950 |
| KJ156356 | LC411995 | KX768207 | FR796488 | HE572972 |
| MG838806 | LC013457 | KX768164 | FR796492 | HE572980 |
| HE572960 | HE572959 | HE572954 | HE572966 | HE572962 |
| HE572942 |  |  |  |  |
