# Supplementary figures and images for: Molecular epidemiology and clinical characteristics of herpangina children in Beijing, China: a surveillance study
Source: PeerJ. 2020 Oct 15;8:e9991. doi: 10.7717/peerj.9991 (PMC7568857; doi:10.7717/peerj.9991)

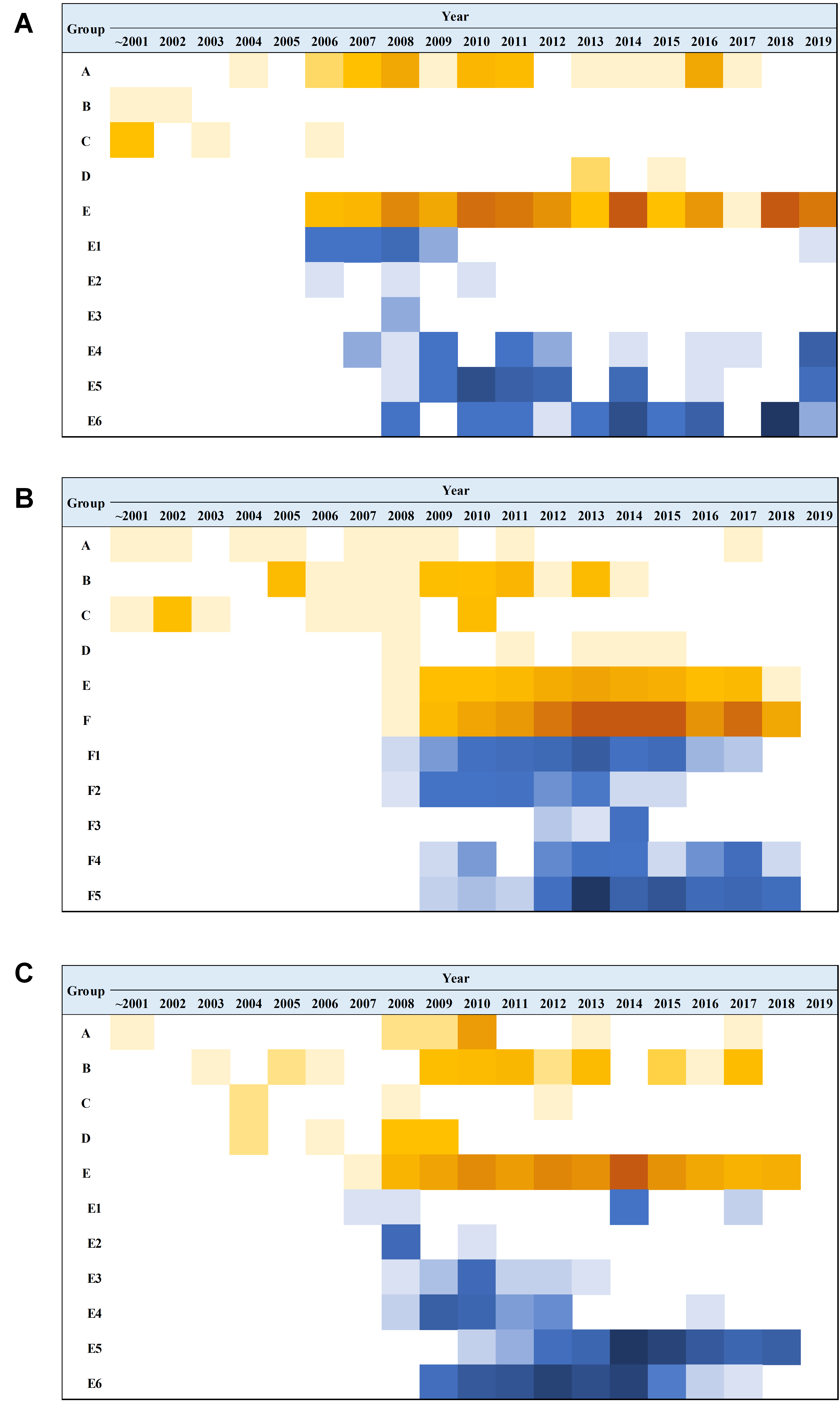

Supplement: Supplemental Information 3 — The shade of color in the figure represented the number of detected sequences for the group in that year. The dark color indicated a large number of sequences, and the light color represented a low number of sequences. A: The temporal distribution of CV-A6; B: The temporal distribution of CV-A4; C: The temporal distribution of CV-A10. [file peerj-08-9991-s003.png]
